# Comparison of EDC/NHS and Pentasodium Triphosphate (TPP) as Cross-Linking Agents for Nanohydroxyapatite, Silk Fibroin, and Chitosan 3D Scaffolds

**DOI:** 10.3390/polym18131610

**Published:** 2026-06-28

**Authors:** Anna Tuwalska, Alina Sionkowska, Grzegorz Tylko, Maciej Przybyłek, Anna Maria Osyczka, Iwona Białas, Michele Laus

**Affiliations:** 1 Department of Biomaterials and Cosmetics Chemistry, Faculty of Chemistry, Nicolaus Copernicus University in Toruń, 87-100 Toruń, Poland; 2 Institute of Advanced Studies, Nicolaus Copernicus University in Toruń, Wileńska 4, 87-100 Toruń, Poland; m.przybylek@cm.umk.pl; 3 Department of Biology and Cell Imaging, Institute of Zoology and Biomedical Research, Faculty of Biology, Jagiellonian University, 30-387 Kraków, Poland; grzegorz.tylko@uj.edu.pl (G.T.); anna.osyczka@uj.edu.pl (A.M.O.); 4 Department of Physical Chemistry, Pharmacy Faculty, Collegium Medicum in Bydgoszcz, Nicolaus Copernicus University in Toruń, Kurpińskiego 5, 85-096 Bydgoszcz, Poland; 5 CosmetoSAFE Consulting LLC, 05-500 Piaseczno, Poland; i.bialas@cosmetosafe.pl; 6 Department of Science and Technological Innovation, University of Eastern Piedmont “A. Avogadro”, 15121 Alessandria, Italy; michele.laus@uniupo.it

**Keywords:** toxicity, cross-linking agents, scaffolds, cellular responses, pore size and shape, silk fibroin, chitosan, nanohydroxyapatite

## Abstract

The present study compares two scaffold cross-linking strategies, EDC/NHS and pentasodium triphosphate (TPP), applied to nanohydroxyapatite/silk fibroin/chitosan (nHA/SF/CTS) scaffolds. The effects of the cross-linking strategy on scaffold morphology, ATR-FTIR profile, swelling, in vitro degradation, and compressive properties were evaluated. In addition, the biological response of selected 20:40:40 and 15:70:15 (*w*/*w*) scaffold variants was assessed using SaOS-2 cells. The IR spectra of TPP- and EDC/NHS-cross-linked materials were qualitatively similar, and no distinct bands attributable to residual EDC or NHS were observed after washing. SEM observations indicated that the TPP-cross-linked scaffolds tended to have smaller and more spherical pores, whereas the EDC/NHS-cross-linked materials showed a more elongated pore morphology. These qualitative morphological differences were accompanied by differences in swelling, degradation, and low-strain mechanical response. The 20:40:40 formulation cross-linked with EDC/NHS showed the most favorable low-strain response, whereas PBS swelling markedly reduced the early compressive response of all scaffolds. Biological assessment showed that the 20:40:40 scaffolds supported viable SaOS-2 populations and maintained osteogenic activity, although cell colonization remained lower than on the control surfaces. The 15:70:15 variant performed less favorably, particularly after TPP cross-linking. These findings indicate that both cross-linking strategies can be used to obtain nHA/SF/CTS scaffolds, but their effects depend strongly on scaffold composition and on the property being considered.

## 1. Introduction

Bone tissue engineering has developed from simple scaffold-based systems toward more advanced in vitro platforms designed to reproduce selected features of the bone microenvironment and improve translational relevance. Despite this progress, scaffolds remain central to this field because they provide a temporary three-dimensional matrix for cell attachment, proliferation, differentiation, extracellular matrix deposition, and tissue organization [1].

Biopolymer-based scaffolds are widely investigated for tissue regeneration because natural polymers can provide biocompatibility, biodegradability, processability, and support for cellular responses. In multicomponent biomaterials, including systems that combine organic polymers with inorganic phases such as hydroxyapatite, the final performance depends not only on the individual components but also on their ratio, network stabilization, and processing history [2]. These advantages are limited when scaffolds dissolve, lose structural integrity, or degrade too rapidly in physiological media. Cross-linking is therefore used to improve scaffold stability, handling resistance, swelling and degradation behavior, and mechanical performance [3]. Because these materials are developed for biomedical applications, the selected cross-linking strategy should also be compatible with the expected biological contact and with the risk-based principles of biological evaluation described in the ISO 10993 framework [4].

**Figure 1:**
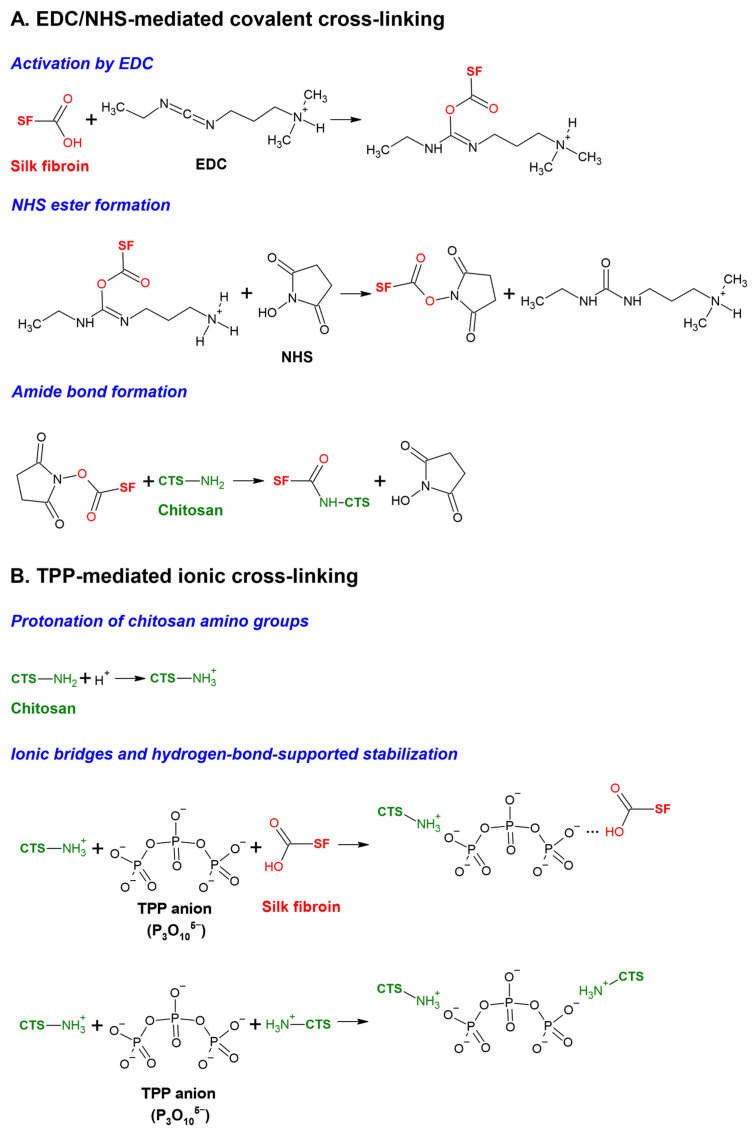
Proposed stabilization routes in nHA/SF/CTS scaffolds cross-linked with EDC/NHS or TPP. (**A**) EDC/NHS-mediated covalent cross-linking through carboxyl-group activation, NHS ester formation, and subsequent amide-bond formation between SF and CTS segments. (**B**) TPP-mediated ionic cross-linking through electrostatic interactions between protonated amino groups of CTS and phosphate groups of the TPP anion, supported by hydrogen bonding with SF and CTS segments. SF and CTS denote silk fibroin and chitosan, respectively.

Numerous chemical agents can be used for the cross-linking of biopolymers. Glutaraldehyde (GA) is a particularly common chemical cross-linker for amino group-containing macromolecules [5,6,7]. A significant limitation of GA is its cytotoxicity, especially when residual amounts remain trapped within the material after processing [5,6]. This makes it difficult to completely exclude adverse tissue reactions associated with this reagent. Other experimentally validated cross-linking systems are therefore widely considered for both protein- and polysaccharide-based biomaterials. A prominent example is EDC/NHS, a carbodiimide-based system that promotes amide-bond formation between carboxyl and amino groups in biopolymer matrices (Figure 1). This system is widely used in the fabrication and chemical cross-linking of collagen- and other biopolymer-based biomaterials because EDC/NHS-mediated activation enables amide-bond formation between carboxyl and primary amino groups, while EDC acts as a zero-length coupling reagent and is not incorporated as a permanent spacer in the final network [8,9,10]. The aqueous solubility of EDC and the formation of water-soluble urea derivatives/by-products can facilitate the removal of unreacted reagents and reaction products during post-cross-linking washing [8,10,11]. However, EDC/NHS cross-linking is not inherently free of biological risk because high cross-linker concentrations, insufficient washing, or carbodiimide-derived intermediates and by-products may impair cell attachment, proliferation, viability, or morphology [8,11,12]. Recent reviews of cross-linked chitosan-based scaffolds also emphasize that the effects of cross-linking should be considered broadly because the selected route can influence mechanical strength, degradation, pore architecture, and biological performance rather than acting only as a simple stabilization step [3,13].

The search for novel and non-toxic cross-linking agents consistent with green chemistry principles therefore remains highly important. Accordingly, increasing attention has been directed toward less toxic cross-linking alternatives, including citric acid, naturally derived agents such as genipin and polyphenolic compounds, and tripolyphosphate (TPP) for ionically cross-linked chitosan-based systems [14,15,16]. Pentasodium triphosphate (Na_5_P_3_O_10_, TPP, Figure 1) is an approved non-toxic food additive commonly used in the dairy industry [17,18]. It functions primarily as an emulsifier in cheese production and regulates the stiffness and rheological properties of cheese-based sauces. These properties arise from its interactions with calcium phosphate nanoclusters, which may induce their dissociation and thereby enhance stabilization of casein micelles [18]. In biomedical applications, TPP is most often used as a cross-linking agent in chitosan-containing nanoparticulate, hydrogel, and scaffold systems, where stabilization relies mainly on ionic rather than covalent interactions. This stabilization is commonly attributed to electrostatic interactions between tripolyphosphate anions and protonated amino groups, with hydrogen bonding also contributing to the final network structure [16,19,20]. Recent reviews further indicate that TPP and other non-covalent or ionic cross-linking routes are relevant in chitosan-based bone scaffold design because they can affect swelling, degradation, mechanical integrity, and biological response in a composition-dependent manner [3,13]. Notably, TPP has also been used in chitosan-based biomaterials, including chitosan and fibroin macromolecular systems intended for nerve regeneration and hemostatic applications [21]. Furthermore, Zhou et al. demonstrated that a composite scaffold comprising silk fibroin, chitosan, and nanohydroxyapatite (SF/CS/nHA) combined with autologous concentrated growth factor significantly promoted proliferation and osteogenic differentiation of bone marrow mesenchymal stem cells while accelerating healing of critical bone defects [22]. The current manuscript also follows this broader line of research by comparing two different cross-linking strategies within an nHA/SF/CTS scaffold system.

The aim of this study was to prepare nHA/SF/CTS porous scaffolds using two cross-linking systems, EDC/NHS and TPP, and to evaluate how the cross-linking route and scaffold composition affect their structure, pore architecture, hydration behavior, degradation, and mechanical performance. The 20:40:40 and 15:70:15 formulations were selected deliberately on the basis of earlier work on nHA/SF/CTS scaffolds, in which these compositionally distinct systems showed favorable biological performance within a broader set of tested ratios [23]. Their inclusion therefore enabled the comparison of covalent and ionic cross-linking in two previously characterized scaffold matrices differing in mineral and biopolymer balance. This formulation-oriented approach is consistent with the development strategy commonly used for SF/CS/nHA composite scaffolds, where the final material response is shaped by both the component ratio and the stabilization method [24]. This view is also consistent with recent hydroxyapatite/chitosan scaffold studies showing that scaffold composition, degradation behavior, and mineral phase organization remain key variables in designing materials for bone repair [25]. A further objective was to relate these material properties to the in vitro response of SaOS-2 cells, including metabolic activity, cell morphology, scaffold colonization, and osteogenic activity. Together, this design was intended to clarify how the selected cross-linking systems affect the balance between structural stability, functional performance, and bone-related cellular response in compositionally distinct nHA/SF/CTS scaffold matrices.

## 2. Materials and Methods

### 2.1. Materials

*Bombyx mori* cocoons used for silk fibroin (SF) isolation were graciously provided by the President of “Polish Silk Ltd.”, Milanówek, Poland. Low molecular weight chitosan (CTS) was provided by the Sigma–Aldrich Company (Poznań, Poland). Both the deacetylation degree (80%) and molecular weight (1.9 × 10^5^ g·mol^−1^) of chitosan were determined using established methods described in the literature [26,27].

Hydroxyapatite in the form of solid powder with particles smaller than 200 nm was provided by the Sigma-Aldrich Company (Poznań, Poland). It was selected intentionally for its nanostructured characteristics, as studies suggest that nanostructured hydroxyapatite (nHA) exhibits superior bioactivity compared to larger-grained forms [28,29,30,31]. Na_2_CO_3_, NaOH, ethanol, and methanol were supplied by the Chempur Company (Piekary Śląskie, Poland). 1-(3-dimethylaminopropyl)-3-ethylcarbodiimide hydrochloride (EDC) and N-hydroxysuccinimide (NHS) were obtained from the Sigma-Aldrich Company (Poznań, Poland). Type I collagen (ColI) derived from bovine skin was purchased from Collado (Brno, Czech Republic). Native chicken egg white lysozyme was obtained from Merck Millipore, Calbiochem^®^, Darmstadt, Germany (Cat. No. 4403-M, ≥20,000 Shugar units mg^−1^ dry weight).

### 2.2. Fabrication of the Scaffolds

CTS and SF solutions were combined in appropriate concentrations and proportions with the addition of nHA (as described previously [32]). Following the procedures reported in the literature [33,34] and the approach originally described by Ajisawa [35], the cocoons were degummed by boiling in 0.5% aqueous Na_2_CO_3_ for 1 h. After filtration, the fibroin solution was dialyzed against distilled water for three days to obtain an aqueous SF solution. Its final concentration was adjusted to 5%, as verified gravimetrically from the dry residue remaining after solvent removal. The CTS solution was prepared separately by dispersing 5 wt% chitosan in 0.5 M acetic acid. The mixture was stirred mechanically and maintained at 40 °C until complete dissolution, which required 48 h and included an overnight pause. Subsequently, an equal volume of SF solution of identical concentration was introduced to CTS solution, followed by nHA addition to prepare the final nHA/SF/CTS mixtures with weight ratios of 20:40:40 and 15:70:15.

**Figure 2:**
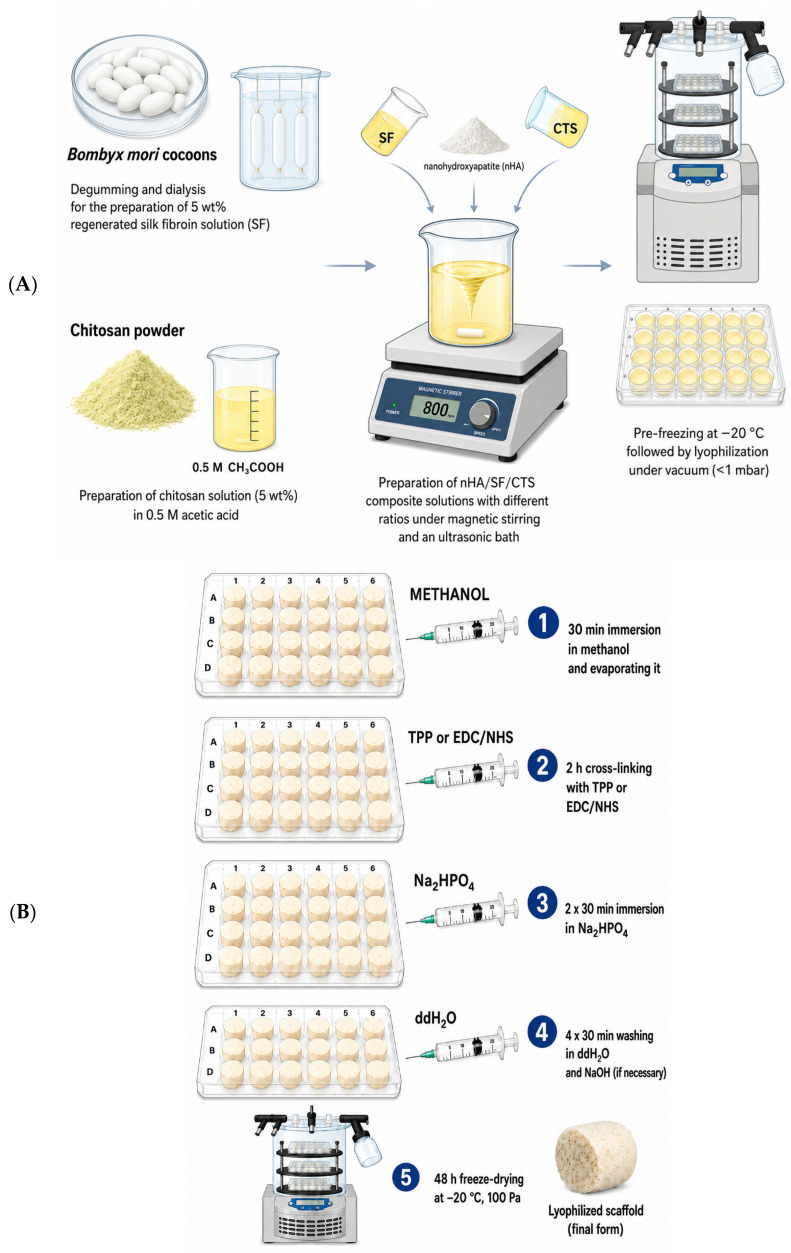
Schematic workflow for the preparation of nanohydroxyapatite/silk fibroin/chitosan (nHA/SF/CTS) scaffolds. (**A**) Preparation of regenerated silk fibroin and chitosan solutions, incorporation of nHA, homogenization, freezing, and first lyophilization. (**B**) Post-treatment of the lyophilized scaffolds, including methanol treatment, cross-linking with TPP or EDC/NHS, washing and neutralization when necessary, and final lyophilization.

The three-component mixtures were homogenized using a magnetic stirrer for 4 h and then sonicated for 15 min in an ultrasonic bath operating at 40 kHz with an effective ultrasonic power of 140 W (Sonic-6, Polsonic, Warsaw, Poland). After homogenization and sonication, the prepared mixtures were transferred to 24-well polystyrene culture plates and kept at −20 °C overnight to induce freezing. The frozen samples were subsequently freeze-dried for 48 h using an Alpha 1-2 LDplus freeze dryer (Martin Christ Gefriertrocknungsanlagen GmbH, Osterode, Germany). The lyophilized samples were carefully removed from the wells and immersed in methanol for 30 min to induce structural ordering of SF and enhance stability in aqueous media. After methanol evaporation at room temperature, the scaffolds were divided into two groups and cross-linked using either TPP or EDC/NHS. For ionic cross-linking, the scaffolds were immersed in a 5% (*w*/*v*) aqueous TPP solution at room temperature for 2 h, using 8–10 mL of cross-linking solution per scaffold to ensure complete immersion in a 24-well plate. For carbodiimide-mediated cross-linking, following the previously reported procedure [36], the scaffolds were immersed for 2 h in an EDC/NHS solution prepared in ethanol at an EDC:NHS molar ratio of 2:1. The concentrations of EDC and NHS were 25 mM and 12.5 mM, respectively, and 8–10 mL of solution was used per scaffold. After cross-linking, the scaffolds were washed twice with 0.1 M Na_2_HPO_4_ solution, using 8–10 mL per scaffold for 30 min each time, and then washed four times with deionized water for 30 min to remove residual reagents and reaction by-products. When necessary, the scaffolds were neutralized with 0.1 M NaOH to eliminate residual acidity originating from the chitosan solvent and then thoroughly rinsed with deionized water. The resulting scaffolds were frozen at −20 °C and subjected to a second lyophilization cycle under a pressure below 100 Pa, approximately 1 mbar, for 48 h, as shown schematically in Figure 2.

For comparative measurements, ColI, SF-only, and CTS-only control materials were prepared. The ColI solution was obtained by dissolving 0.5% commercially available bovine skin collagen in water and was cross-linked using the EDC/NHS system according to Slovikova et al. [37]. SF-only and CTS-only materials were prepared analogously, cross-linked with either EDC/NHS or TPP, and processed under the same drying and post-treatment conditions as the composite scaffolds. The lyophilized samples were then examined using the methods described below.

### 2.3. ATR-FTIR Spectroscopy

Fourier transform infrared (FTIR) spectra of the scaffolds were recorded using a Genesis II spectrophotometer (Mattson, Fremont, CA, USA) coupled with a MIRacle™ attenuated total reflectance (ATR) attachment (PIKE Technologies, Fitchburg, WI, USA) with a ZnSe crystal. Spectra were acquired in absorbance mode using 64 scans at a resolution of 4 cm^−1^.

### 2.4. Scanning Electron Microscopy (SEM) of the Cross-Linked Materials

The morphological features of final cross-linked nHA/SF/CTS scaffolds with 20:40:40 and 15:70:15 (*w*/*w*) ratios were examined using scanning electron microscopy (SEM) (LEO Electron Microscopy Ltd., Cambridge, UK). Prior to imaging, the scaffolds were frozen in liquid nitrogen for 3 min and then fractured to obtain fresh cross-sections for SEM observation of the internal pore architecture. The fractured materials were sputter-coated with a thin gold-palladium (Au/Pd) alloy layer using an SC7620 sputtering device (Quorum Technologies, Laughton, UK).

### 2.5. Swelling Test

The swelling behavior of the scaffolds was evaluated in phosphate-buffered saline (PBS, pH 7.4) at 37 °C. Lyophilized samples of SF, CTS, and nHA/SF/CTS scaffolds cross-linked with EDC/NHS or TPP were first weighed in the dry state (*W*_0_) and then immersed in PBS. At predefined time points (15, 30, 45, and 60 min), the samples were removed, gently blotted with filter paper to eliminate excess liquid from the surface, and weighed immediately in the swollen state (*W_t_*). The swelling ratio was calculated according to Equation (1):

(1) $$Swelling\left(\%\right)=\frac{W_{t}-W_{0}}{W_{0}}\times 100$$

where *W*_0_ is the initial dry weight of the sample and *W_t_* is the sample weight after immersion for time *t*. All swelling experiments were performed in triplicate. Representative macroscopic photographs of the samples were also collected during the swelling experiment.

### 2.6. In Vitro Degradation Test

The in vitro degradation test was carried out using 1.5 cm × 0.9 cm samples of the investigated materials immersed in 5 mL of phosphate-buffered saline (PBS, pH 7.4) at 37 °C in the presence of native chicken egg white lysozyme (Merck Millipore, Calbiochem^®^, Cat. No. 4403-M, ≥20,000 Shugar units mg^−1^ dry weight) at a concentration of 1.5 μg mL^−1^ for 49 days. The lysozyme concentration was selected to approximate physiological conditions reported in the literature for chitosan degradation studies [38,39]. The lysozyme solution was refreshed daily to maintain consistent enzyme activity, and scaffolds were rinsed with distilled water. The extent of in vitro degradation of the materials was determined by measuring their weight loss (Equation (2)):

(2) $$Weight\;loss(\%)=\frac{W_{0}-W_{t}}{W_{0}}\times 100$$

where *W*_0_ and *W_t_* denote the dry weight before the degradation and the dry weight after time *t*, respectively. Control samples were incubated under the same conditions but without lysozyme. All in vitro degradation tests were carried out in triplicate.

### 2.7. Compression Testing

The mechanical properties of the nHA/SF/CTS scaffolds were evaluated in compression using a Zwick&Roell 0.5 testing machine (Zwick&Roell Group, Ulm, Germany). The specimens used for compression testing had a cylindrical geometry. Before testing, the dimensions of each specimen were recorded and entered into the testing software for stress–strain calculation.

Compression tests were performed with reference to ISO 604 for the determination of compressive properties [40]. Dry specimens were tested at room temperature, whereas hydrated specimens were tested in a PBS-containing chamber (pH 7.4, 37 °C). A crosshead speed of 2 mm/min was used for dry specimens, whereas 10 mm/min was used for hydrated specimens. Before each measurement, a small compressive preload of 0.05 N was applied to establish stable contact between the specimen and the compression plates and to reduce specimen displacement during testing. Measurements were performed in five replicates for each scaffold group. Full compressive stress–strain curves recorded under dry and swollen conditions are provided in the Supplementary Materials as Figures S1–S8.

For all scaffold specimens, two low-strain mechanical descriptors were extracted: the initial compressive modulus, E, and the compressive stress at 1% strain, σ_1%_. The initial compressive modulus was calculated from the slope of the initial linear segment within a strain range of 0.05% to 2%. The σ_1%_ parameter was defined as the compressive stress recorded at 1% strain. These low-strain descriptors were selected to characterize early scaffold stiffness and resistance before pronounced pore-wall rearrangement and structural compaction.

### 2.8. In Vitro Biocompatibility Assessment

SaOS-2 osteosarcoma cells (89050205-1VL, Merck, Darmstadt, Germany) were used as a model to assess the biocompatibility of the selected nHA/SF/CTS scaffolds. First, the samples of freeze-dried material were sterilized with UV radiation for 10 min (both sides) inside the laminar flow chamber (Safemate EZ 1.2, EuroClone, Pero, Italy). A sterile 10 mm biopsy punch was used to excise discs from all biomaterials. Then, the discs were separately placed inside the wells of a 48-well plate (Wuxi NEST Biotechnology Co., Ltd., Wuxi, China) and gently pressed with the polypropylene rings to the bottom of the wells to protect the discs from floating in culture media. As a reference, Thermanox™ coverslips (#174950, Thermo Fisher Scientific, Waltham, MA, USA) were placed in wells without biomaterial scaffolds and held with the same polypropylene rings. Finally, all wells were moistened with PBS for 1 h. All experiments were performed in triplicate.

SaOS-2 cells were routinely cultured in T75 flasks (Wuxi NEST Biotechnology Co., Ltd., Wuxi, China) until 90–95% confluence. Next, the cells were seeded onto each biomaterial disc as well as directly on the Thermanox™ surface (a blank reference) at a density of 2 × 10^3^ cells cm^−2^. SaOS-2 cells were cultured for one week in a cell culture incubator (CCL-170B-8 CelCulture^®^, ESCO, Singapore), and the medium was replaced with fresh medium every three days. All assays were performed in triplicate.

### 2.9. Proliferation/Metabolic Activity of SaOS-2 Cultured on Cross-Linked Materials

After one week of culture, SaOS-2 cells were thoroughly washed with PBS to remove residual medium from the porous structure of the discs and covered with fresh MTS assay solution (CellTiter 96^®^ AQueous One Solution Cell Proliferation Assay, Promega, Madison, WI, USA) prepared according to the manufacturer’s protocol. The culture plate was incubated for 1 h, after which the MTS solution was transferred from the specimens to separate wells of a 96-well plate. The absorbance of the MTS solution was measured using a microplate reader at 492 nm (SpectraMax iD3, Molecular Devices, San Jose, CA, USA), and the results were normalized to the mean value of the Control group.

### 2.10. Differentiation Potential of SaOS-2 Cells Cultured on Cross-Linked Materials

After one day of culture, the medium was supplemented with 100 μg·mL^−1^ ascorbate 2-phosphate (ASC-2P) and 10 µg·L^−1^ dexamethasone (Dex) to induce osteoblastic differentiation of SaOS-2 cells [41]. The cells were cultured for 7 days under osteogenic conditions, followed by measurement of alkaline phosphatase (ALP) activity according to the method described by Bessey et al. [42]. Briefly, after 7 days of culture, SaOS-2 cells were washed thoroughly with PBS, and their metabolic activity was assessed using the MTS assay, as described above. The cells were then treated with cell lysis buffer to extract ALP. Next, the extract was added to a colorless p-nitrophenyl phosphate (pNPP) solution to allow active ALP to hydrolyze the substrate into a yellow product, p-nitrophenol. The enzyme activity was monitored in kinetic mode by means of a microplate reader at 405 nm (SpectraMax iD3, Molecular Devices, San Jose, CA, USA), and the results were compared to the standard curve developed from increasing concentrations of p-nitrophenol (nM). Finally, the results obtained were normalized to the MTS data.

### 2.11. Scanning Electron Microscopy (SEM) of SaOS-2 Cultured on Cross-Linked Materials

After a week of SaOS-2 culture on the surface of the cross-linked materials, the discs with cells were washed three times with PBS and fixed for 1 h with a mixture of freshly prepared 2% formaldehyde/2% glutaraldehyde in PBS solution. Then, the biomaterials were thoroughly washed with PBS and dehydrated with increasing concentrations of ethanol. The discs of biomaterials were dried using a critical point dryer (CPD, E3100, Quorum Technologies, Laughton, UK), gently attached to specimen holders with carbon adhesive glue (PELCO, Agar Scientific, Rotherham, UK), and finally coated with a thin layer of gold (JFC-1100E, JEOL, Tokyo, Japan). A JEOL JSM-5410 (JEOL, Tokyo, Japan) scanning electron microscope was used to image all specimens at an accelerating voltage of 15 keV in secondary electron detection mode.

### 2.12. Statistical Analysis

To compare the experimental groups, a one-way ANOVA was performed, followed by Tukey’s HSD post hoc test. All statistical analyses were conducted using Statistica 13.1 software (TIBCO Software Inc., Palo Alto, CA, USA) [43]. Differences were considered statistically significant at *p* < 0.05.

## 3. Results and Discussion

### 3.1. The Toxicological Characteristics of Applied Cross-Linking Agents

This study examined two widely used cross-linking systems, TPP and EDC/NHS, in the context of biocompatible silk-based scaffolds for bone regeneration. TPP is an inorganic salt, whereas EDC is an organic carbodiimide. These fundamental differences affect not only the properties of the materials synthesized using these agents but also their impacts on human health and the environment. A summary of the key experimental and calculated toxicological data for TPP and EDC is presented in Table 1. Notably, NHS has been used in greener synthetic systems and biomedical functionalization workflows [44,45]. In this context, NHS is not expected to be a major source of residual toxicity after adequate washing [46].

TPP showed no oral acute toxicity, no eye irritation, negative skin sensitization, and no genotoxicity in both in vivo and in vitro studies. It also shows no carcinogenicity, mutagenicity, or reproductive (CMR) toxicity with a No Observed Effect Level (NOEL) parameter in diet equivalent to 0.5% according to a repeated dose toxicity test (2-year, oral, rat). For EDC, data on acute toxicity are lacking. Similarly to TPP, it does not exhibit eye irritation. However, EDC shows positive skin sensitization and selected in vitro genotoxicity alerts, whereas available regulatory information does not support its classification as a germ-cell mutagen, carcinogen, or reproductive toxicant.

### 3.2. ATR-FTIR Analysis of Obtained Materials

**Figure 3:**
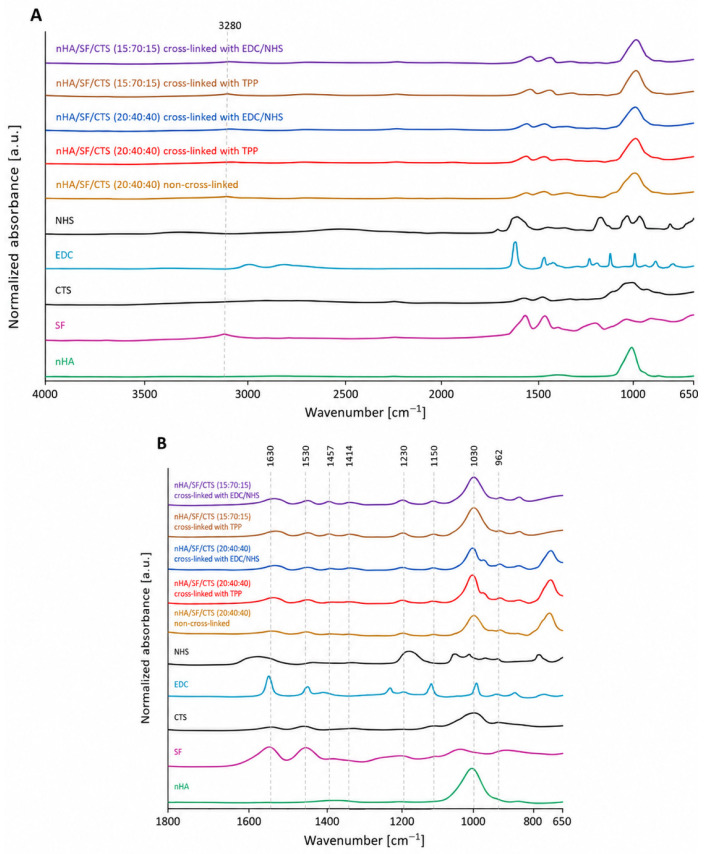
ATR-FTIR spectra of nHA/SF/CTS scaffolds and reference materials. (**A**) Full spectral range. (**B**) Expanded fingerprint region from 1800 to 650 cm^−1^ with selected band positions marked to facilitate peak comparison. The spectra include individual scaffold components (nHA, SF, and CTS), reference spectra for EDC and NHS, the non-cross-linked nHA/SF/CTS (20:40:40) scaffold, and nHA/SF/CTS scaffolds cross-linked with TPP or EDC/NHS at 20:40:40 and 15:70:15 weight ratios. Spectra were normalized independently and vertically offset for clarity. Peak positions are shown as wavenumbers in cm^−1^.

The ATR-FTIR spectra of the prepared materials confirmed the presence of the main scaffold components and allowed comparison of how composition and cross-linking affected the spectral profiles of the nHA/SF/CTS systems (Figure 3). Infrared spectroscopy has long been used to analyze silk fibroin structure, including spectral features related to its secondary structure and their relationship to material organization and properties [49,50,51]. All composite spectra showed a broad band with a maximum near 3280 cm^−1^, attributable to overlapping O–H and N–H stretching vibrations. The spectra also contained the characteristic amide region of the organic phase, with amide I at 1625–1630 cm^−1^, amide II at 1529–1535 cm^−1^, and weaker amide III contributions at 1230–1265 cm^−1^. These band positions agree with previous reports for fibroin- and chitosan-containing materials. The band positions also support a β-sheet contribution in the fibroin phase, which is consistent with the methanol treatment applied before cross-linking [52,53]. However, ATR-FTIR alone does not allow reliable quantification of fibroin secondary structure in multicomponent composites. The weak feature near 1230 cm^−1^ in the non-cross-linked material may reflect a less ordered or random-coil contribution, as reported in earlier infrared studies of silk fibroin [53,54].

Both scaffold compositions, 20:40:40 and 15:70:15, showed the same general set of absorption bands. Materials cross-linked with TPP and EDC/NHS also showed similar overall spectral patterns. These results indicate that, within the investigated compositional range, changing the component ratio or cross-linking route did not introduce new functional groups detectable by ATR-FTIR. The spectral differences were mainly limited to relative band intensities and band overlap. Such variations are expected in ternary systems containing different proportions of silk fibroin, chitosan, and nanohydroxyapatite.

The spectrum of pure CTS showed absorption bands typical of commercial chitosan, including a broad band in the 3500–3000 cm^−1^ region and amide-related signals near 1630 and 1530 cm^−1^ [55,56]. These signals can be attributed to residual N-acetyl groups associated with incomplete deacetylation [57,58,59]. Pure nHA showed the expected phosphate vibrations in the 800–1150 cm^−1^ range, while the weak bands near 1414 and 1457 cm^−1^ may be associated with carbonate-related species. These mineral-related bands were also present in all nHA/SF/CTS spectra, confirming the presence of nHA in the composite structure. At the same time, signals in the 1400–1500 cm^−1^ range should be interpreted with caution, since they do not by themselves provide unambiguous evidence for a specific mode of carbonate substitution in hydroxyapatite [60,61].

The 1000–1250 cm^−1^ region requires particularly careful interpretation because it contains overlapping contributions from phosphate vibrations of nHA, C–O–C vibrations of CTS, and amide III bands of SF. Therefore, the small feature observed near 1150 cm^−1^ is better treated as part of an overlapping spectral region rather than assigned exclusively to a single vibrational mode. A similar caution applies to the TPP-cross-linked materials, since the characteristic phosphate bands of TPP may be difficult to distinguish unambiguously in the composite spectra because of overlap with strong absorptions from nHA and the organic matrix [62,63].

Importantly, the spectra of the composites cross-linked with TPP and EDC/NHS remained qualitatively similar. No distinct additional bands that could be unequivocally assigned to residual EDC or NHS were observed after washing. This is consistent with the fact that EDC/NHS coupling forms direct amide-type linkages between existing functional groups and does not introduce a permanent linker-derived moiety into the final network [64].

### 3.3. Swelling Analysis

The swelling behavior is an important functional property of cross-linked scaffolds intended for biomedical applications because it affects fluid uptake, internal wetting, and the transport of dissolved species throughout the material [65,66,67]. Previous studies on silk fibroin/chitosan systems have shown that the incorporation of chitosan can enhance water retention and substantially modify the hydration behavior of fibroin-based materials [32,38,68,69].

**Figure 4:**
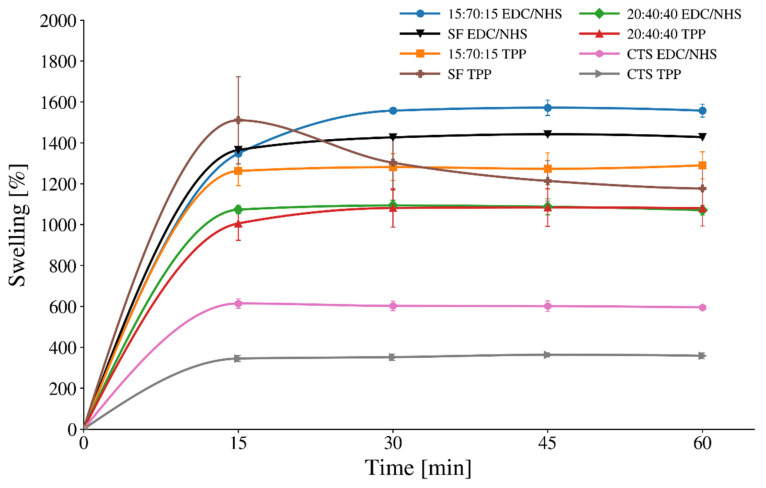
Swelling ratio of nHA/SF/CTS scaffolds with weight ratios 20:40:40 and 15:70:15 in PBS after cross-linking with EDC/NHS or TPP as a function of time. For comparison, the corresponding cross-linked SF and CTS samples are also shown.

In the present study, swelling experiments were carried out in PBS at 37 °C and pH 7.4 to approximate physiological conditions. At this pH, chitosan is much less soluble than in acidic media, where protonation of amino groups promotes chain solubilization [70]. Therefore, the higher swelling of selected scaffolds should not be interpreted as dissolution-driven swelling of the chitosan phase. In these porous composites, water uptake reflects the combined contribution of the SF-rich matrix, the chitosan-containing network, accessible pore volume, inorganic phase distribution, and cross-linking chemistry. This interpretation is consistent with recent analyses of ternary biomaterials and chitosan-based biocomposite scaffolds, where swelling and degradation are described as composition- and cross-linking-dependent properties rather than as functions of a single polymer phase [2,3]. In TPP-cross-linked systems, ionic interactions between phosphate groups and protonated amino groups, together with hydrogen bonding, can restrict chain mobility and reduce water uptake. Such effects are known to depend on pH, ionic environment, and cross-linking density in chitosan/TPP systems [19,20,27,71]. As shown in Figure 4, most samples exhibited a rapid increase in swelling during the first 15 min and then approached an apparent plateau, with only minor changes at longer times. A similar time scale was reported previously for SF/CTS sponges without nHA, which suggests that the addition of nHA did not markedly change the overall rate of liquid uptake in these systems [32,38]. Among the composite scaffolds, the 15:70:15 formulation cross-linked with EDC/NHS showed the highest swelling ratio. The corresponding 15:70:15 scaffold cross-linked with TPP showed lower values but still swelled more than the two 20:40:40 composites. The swelling ratios of the 20:40:40 materials were clearly lower and remained relatively close to each other. The single-component controls help explain this trend, although the swelling of the composite scaffolds reflects the combined behavior of the SF, CTS, and nHA phases rather than the response of any single component alone. CTS cross-linked with TPP showed the lowest and most stable swelling profile, whereas SF cross-linked with EDC/NHS remained highly swollen throughout the experiment. In contrast, SF treated with TPP showed an early maximum followed by a gradual decrease, which suggests structural relaxation or partial loss of integrity rather than a stable equilibrium swelling state.

These results indicate that the apparent swelling ratio in the investigated scaffolds should not be interpreted solely in terms of cross-link density. In highly porous materials, the measured swelling also reflects how effectively the liquid penetrates and occupies the internal pore volume. Therefore, the greater swelling observed for the EDC/NHS-cross-linked composites is more reasonably attributed to more effective volumetric wetting and better preservation of accessible internal space during immersion. This interpretation is consistent with previous reports showing that SF/CTS and SF/CTS/nHA materials can form porous architectures with interconnected internal networks and that porosity and pore morphology can influence water absorption and fluid transport [69,72,73].

**Figure 5:**
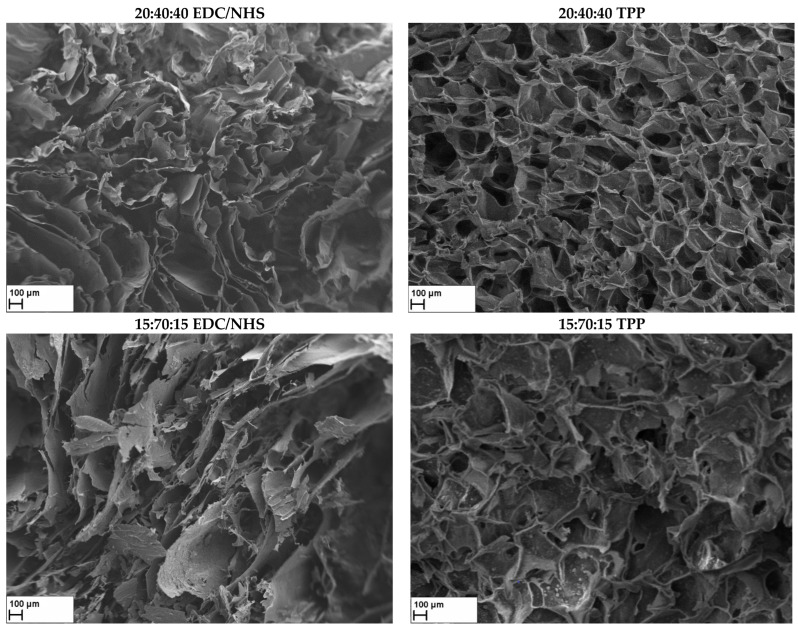
Scanning electron microscopy images of nHA/SF/CTS scaffolds after cross-linking with EDC/NHS or TPP. The upper row corresponds to the 20:40:40 formulation, whereas the lower row corresponds to the 15:70:15 formulation. The (**left**) column shows scaffolds cross-linked with EDC/NHS, while the (**right**) column shows scaffolds cross-linked with TPP. Scale bar: 100 μm.

The SEM images shown in Figure 5 are consistent with this interpretation and indicate qualitative differences in pore morphology between the EDC/NHS- and TPP-cross-linked scaffolds. The porous architecture of related nHA/SF/CTS scaffold formulations, including the 20:40:40 and 15:70:15 systems, was previously characterized by SEM and pore-size analysis, which provided the basis for selecting these compositions for further cross-linking comparison in the present study [23]. In these qualitative SEM observations, the EDC/NHS-cross-linked scaffolds appeared to have thicker lamellar walls and broader internal channels, whereas the TPP-cross-linked scaffolds appeared to show thinner walls and a finer pore system. This contrast was especially evident for the 15:70:15 scaffold, where the TPP-cross-linked material appeared more constricted and less open than its EDC/NHS-cross-linked counterpart. These observations may indicate less effective stabilization of the fibroin-richer scaffold by TPP under the present conditions. By contrast, the 20:40:40 scaffold cross-linked with TPP showed a relatively large number of visible open pores, which may be related to its higher chitosan content. This compositional effect is chemically reasonable because TPP is a well-established ionic cross-linker for chitosan, whereas its stabilizing effect is expected to be less effective in fibroin-richer systems [27,62].

**Figure 6:**
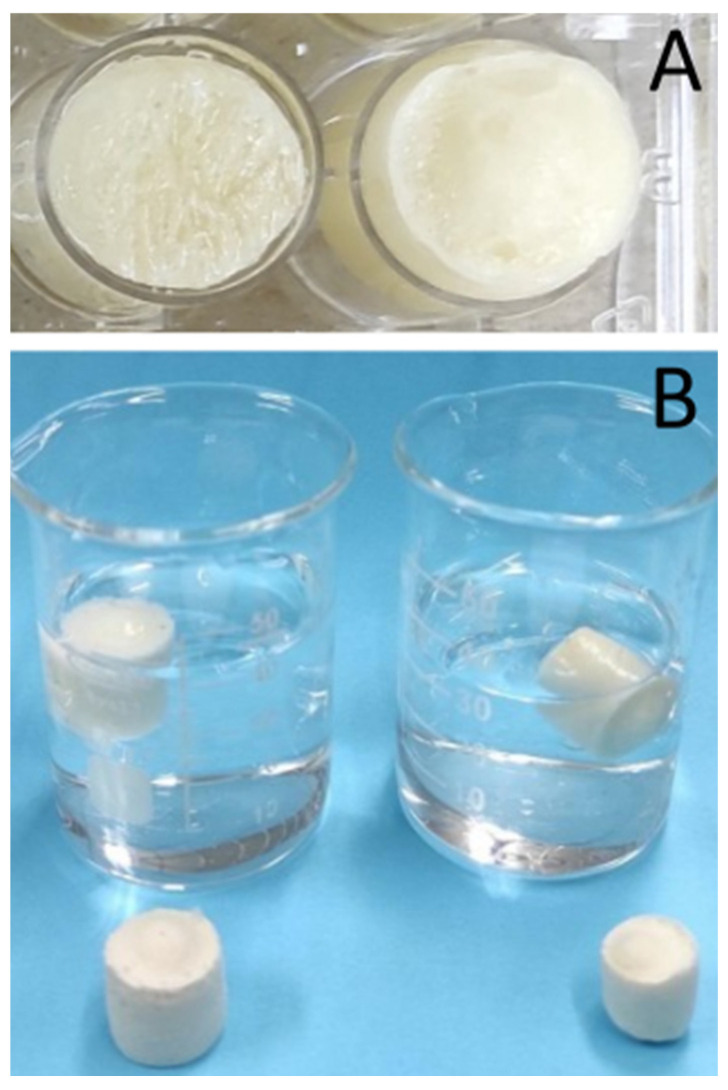
Macroscopic appearance of nHA/SF/CTS (20:40:40) scaffolds before, during, and after the swelling experiment. Behavior of scaffolds cross-linked using different methods (EDC/NHS **left**, TPP-**right**) after immersion in PBS (**A**) in the wells of a 24-well plate (**B**) in a beaker.

The macroscopic photographs collected during the swelling experiment and shown in Figure 6 further indicate that the two cross-linking routes led to different wetting behavior. Together with the SEM observations, these images suggest that the EDC/NHS-cross-linked scaffold can be wetted more uniformly throughout its volume, whereas the TPP-cross-linked scaffold appeared to undergo less complete internal penetration by PBS. In the latter case, hydration may have occurred preferentially in the outer regions of the scaffold, while narrowing of internal pore pathways could have limited further liquid ingress. This explanation is particularly plausible for the 15:70:15 scaffold, for which TPP appears to be a less favorable stabilizing agent under the investigated conditions. Literature on TPP-cross-linked chitosan systems also shows that pore interconnectivity, swelling, and structural homogeneity depend strongly on cross-linking conditions and can change substantially with the extent of ionic cross-linking [27,62].

From the perspective of scaffold performance, these observations are important because pore morphology, pore interconnectivity, and effective internal wetting can influence fluid exchange within the material. These structural features are closely related to nutrient transport, waste removal, and the conditions required for cell survival and colonization within porous scaffolds designed for bone regeneration [65,73,74,75]. In this context, the swelling curves, SEM images, and macroscopic observations consistently indicate that EDC/NHS treatment produced scaffolds capable of more effective volumetric wetting, whereas the behavior of the TPP-cross-linked materials depended more strongly on the initial composition and was less favorable for the fibroin-richer system.

### 3.4. Mechanical Properties

Mechanical performance is a key functional aspect of porous scaffolds intended for bone regeneration because the material must retain sufficient stiffness to provide structural support while remaining permeable to fluids and cells [66,67,76,77]. In the present systems, mechanical behavior has to be considered together with swelling and morphology, since hydration, pore architecture, and interactions between scaffold components jointly determine the compressive response.

**Figure 7:**
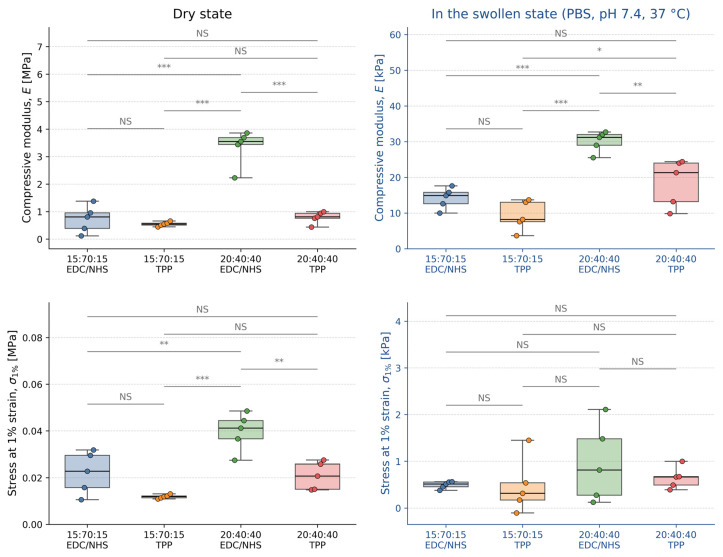
Low-strain compressive properties of nHA/SF/CTS scaffolds with weight ratios of 20:40:40 and 15:70:15 after cross-linking with EDC/NHS or TPP. Compressive modulus, E, and stress at 1% strain, σ_1%_, are shown for dry scaffolds and scaffolds tested in the swollen state in PBS (pH 7.4, 37 °C). Boxes indicate the interquartile range, horizontal lines indicate medians, whiskers indicate minimum and maximum values, and points show individual measurements (*n* = 5). Statistical comparisons were performed using one-way ANOVA followed by Tukey’s HSD post hoc test. NS, not significant, * *p* < 0.05, ** *p* < 0.01, *** *p* < 0.001.

Figure 7 summarizes the low-strain mechanical response of the nHA/SF/CTS scaffolds, including the distribution of measurements and comparisons between the respective groups. The corresponding mean values with standard deviations are listed in Table 2. The full compressive stress–strain curves are provided in the Supplementary Materials as Figures S1–S8. These curves show the compression response recorded for each scaffold group and illustrate the marked change in deformation behavior after PBS swelling. Previous studies on related silk fibroin/chitosan-based materials have shown that their mechanical properties can be tuned by composition and that blending SF with CTS may improve compressive behavior relative to pure fibroin sponges [38,78,79].

In the dry state, the EDC/NHS-cross-linked 20:40:40 scaffold showed the most favorable low-strain mechanical response, with the highest compressive modulus (E) and compressive stress at 1% strain (σ_1%_) among all evaluated groups (Figure 7, Table 2). Both parameters were clearly higher than those of the TPP-cross-linked 20:40:40 scaffold and both 15:70:15 variants. The 15:70:15 formulations generally showed lower compressive resistance, most clearly in comparison with the 20:40:40 scaffold cross-linked with EDC/NHS. Differences among 15:70:15 EDC/NHS, 15:70:15 TPP, and 20:40:40 TPP were smaller and were not consistently significant. The relatively large scatter in E for the dry 15:70:15 EDC/NHS specimens likely reflects structural heterogeneity typical of highly porous materials, where local pore-wall arrangement and load-bearing continuity can affect the initial slope of the stress–strain curve. This trend indicates that the mechanical benefit of EDC/NHS cross-linking was most pronounced in the 20:40:40 formulation. Therefore, the observed response cannot be attributed to cross-linking chemistry alone. It more likely reflects the combined influence of scaffold composition, nHA content, chitosan fraction, and pore-wall architecture.

Swelling in PBS substantially reduced the low-strain mechanical response of all formulations, with E decreasing from the MPa range to the kPa range. This confirms marked softening of the porous matrices under hydrated conditions (Figure 7, Table 2). Nevertheless, the 20:40:40 EDC/NHS scaffold maintained the highest modulus in the swollen state and remained significantly stiffer than the other groups. The 20:40:40 TPP scaffold also showed a higher modulus than the 15:70:15 TPP scaffold, whereas its difference from the 15:70:15 EDC/NHS scaffold was not significant. After swelling, σ_1%_ values were very low in all groups, and no significant differences were observed among the swollen scaffolds. This agrees with the low absolute stress values measured at 1% strain and with the scatter expected during the initial compression of hydrated, highly porous structures. At this early deformation stage, small differences in initial contact, local pore-wall engagement, and collapse of fluid-filled pores may affect σ_1%_, especially when the recorded response is close to the lower force range of the measurement.

Although TPP can reinforce chitosan-based networks through ionic interactions with protonated amino groups, the present data indicate that EDC/NHS-mediated covalent stabilization provided the most favorable modulus response in the 20:40:40 scaffold, both in the dry and swollen states. More broadly, the recent literature on chitosan-based and chitosan-composite scaffolds shows that covalent and ionic cross-linking routes, including EDC/NHS- and TPP-based strategies, can modify compressive behavior, degradation, porosity, and cellular response. However, the direction and magnitude of these effects depend strongly on scaffold composition and processing conditions [13,80].

The pronounced decrease in mechanical response after PBS incubation is consistent with the swelling analysis, which confirmed liquid uptake and internal wetting of the scaffolds. Water acts as a plasticizing medium by increasing polymer chain mobility, weakening non-covalent intermolecular interactions, and reducing the compressive resistance of hydrated pore walls. In porous biomaterials, this effect is further amplified by swelling and internal fluid penetration, which together lower the apparent resistance of the material under compression [81,82,83,84]. At a more fundamental level, the compressive response of silk fibroin has also been linked to the intrinsic mechanical behavior of its crystalline domains under pressure [85].

From a scaffold-design perspective, dry-state stiffness is important for handling and initial structural support, whereas the hydrated response better reflects the aqueous environment in which the scaffold is expected to operate. The full stress–strain curves show that all evaluated scaffolds behave as mechanically soft, porous materials after swelling in PBS. In practical terms, the main distinction among the groups lies in the balance between dry-state stiffness, low-strain compressive resistance, wetting behavior, and internal structural accessibility. This interpretation is consistent with recent work on porous silk-protein scaffolds, where compressive properties and osteogenic response depended on scaffold composition, pore architecture, and mineralization state rather than on a single material parameter alone [86]. In this context, the 20:40:40 formulation shows the more favorable mechanical profile, especially after EDC/NHS cross-linking, whereas the 15:70:15 formulation shows lower resistance to compression.

### 3.5. Degradation Profiles

Controlled degradability is a fundamental requirement for scaffolds intended for bone regeneration because the material must provide temporary support at the defect site and subsequently yield to newly formed tissue. Scaffold degradation should therefore be coordinated with the progression of tissue repair rather than treated as an isolated material parameter [87,88]. Degradation in the present study was evaluated in PBS containing lysozyme. This medium is biologically relevant for chitosan-containing systems because lysozyme is one of the enzymes involved in chitosan cleavage under physiological conditions [89,90].

**Figure 8:**
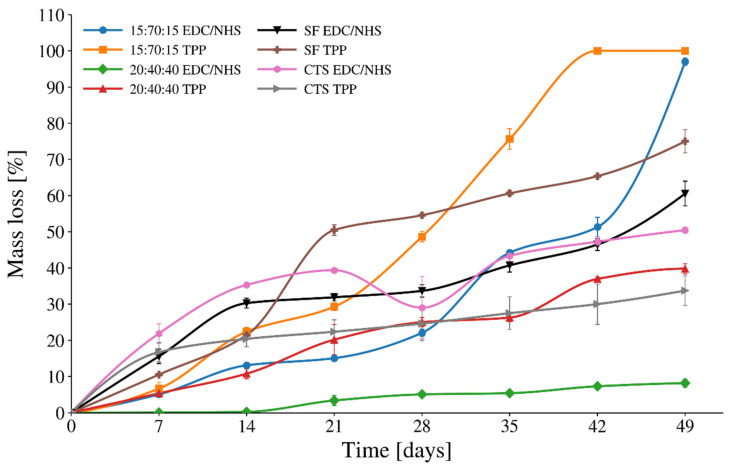
Degradation profile of SF, CTS, and nHA/SF/CTS scaffolds with weight ratios 20:40:40 and 15:70:15 after cross-linking with EDC/NHS or TPP in 1.5 μg mL^−1^ lysozyme solution as a function of time.

The degradation profiles presented in Figure 8 indicate that both scaffold composition and the cross-linking route strongly affected mass loss during incubation. The 20:40:40 material cross-linked with EDC/NHS proved to be the most stable among the composite scaffolds and reached a mass loss of only 8.16 ± 1.10% after 49 days. The corresponding 20:40:40 scaffold cross-linked with TPP degraded more extensively and exhibited a 39.81 ± 1.41% mass loss at the same time point. A distinctly different pattern emerged for the 15:70:15 composition. The scaffold cross-linked with EDC/NHS displayed moderate degradation during the first five weeks before experiencing a sharp increase in mass loss to reach 97.02 ± 1.05% after 49 days. Furthermore, the 15:70:15 scaffold cross-linked with TPP degraded even more rapidly and achieved complete disintegration by day 42. These results show that the degradation behavior of the composite scaffolds cannot be described by a single general rule based exclusively on the identity of the cross-linking agent.

The single-component controls facilitate a clearer understanding of this phenomenon. Pure CTS degraded more substantially when cross-linked with EDC/NHS than with TPP and reached mass losses of 50.46 ± 0.88% and 32.36 ± 1.67% after 49 days, respectively. The opposite trend was observed for pure SF. The TPP-treated SF material degraded more rapidly than the EDC/NHS-treated counterpart and reached mass losses of 75.02 ± 3.21% and 60.56 ± 3.39%, respectively. The final degradation profile of the nHA/SF/CTS composites consequently reflects not only the type of cross-linker used but also its compatibility with the dominant organic component of the scaffold. This observation is chemically reasonable because TPP acts primarily as an ionic cross-linker for chitosan through interactions with protonated amino groups, whereas EDC/NHS stabilizes the network through covalent coupling [27,62]. This difference helps explain why the 15:70:15 scaffold cross-linked with TPP was particularly unstable. In a scaffold containing 70 wt% SF and only 15 wt% CTS, the fraction of the organic phase that can be efficiently stabilized by TPP is likely to be relatively limited. Under such conditions, ionic cross-linking appears insufficient to maintain structural continuity during prolonged immersion and enzymatic exposure, consistent with the rapid disintegration of the 15:70:15 TPP material. The balance between these mechanisms in mixed systems therefore depends strongly on the initial composition.

The composition-dependent nature of this degradation is broadly consistent with our earlier work on related EDC/NHS-cross-linked nHA/SF/CTS scaffold formulations. In that study, the addition of chitosan increased resistance to degradation and extended the degradation time of the materials, while the 20:40:40 formulation was among the most degradation-resistant composite variants [23]. Pure SF and pure CTS degraded more rapidly than selected nHA/SF/CTS composites in that previous work. The present data extend that comparison by showing that the degradation profile is further modulated by the cross-linking route and that this effect depends strongly on the relative amounts of SF and CTS.

The present findings also align with the swelling behavior and mechanical response discussed in Section 3.3 and Section 3.4. The 15:70:15 scaffolds showed higher swelling and generally lower low-strain resistance, with the least favorable overall behavior observed after TPP cross-linking. These features likely increased the accessibility of the scaffold interior to the degradation medium and reduced its resistance to progressive mass loss. The 20:40:40 scaffolds, particularly the EDC/NHS-cross-linked variant, combined lower swelling, higher low-strain modulus, and slower degradation. The degradation behavior therefore appears to be governed by the combined effects of network stabilization, fluid accessibility, and the intrinsic susceptibility of the organic phase to enzymatic attack rather than by the cross-linking chemistry alone.

These differences are highly relevant from the perspective of scaffold design. A very slow degradation rate may prolong structural support, yet excessively persistent scaffolds can delay replacement by newly formed tissue. An overly rapid degradation process may, in turn, compromise structural integrity before regeneration is sufficiently advanced. In this context, the present results suggest that the 20:40:40 scaffold cross-linked with EDC/NHS provides the highest overall stability. The 15:70:15 scaffold cross-linked with TPP, by contrast, appears too unstable to ensure prolonged structural support under the investigated in vitro degradation conditions. The remaining systems occupy an intermediate position and may offer a more balanced compromise between persistence and resorbability depending on the intended application.

### 3.6. In Vitro Characteristics of nHA/SF/CTS Scaffolds—Biological Activity

**Figure 9:**
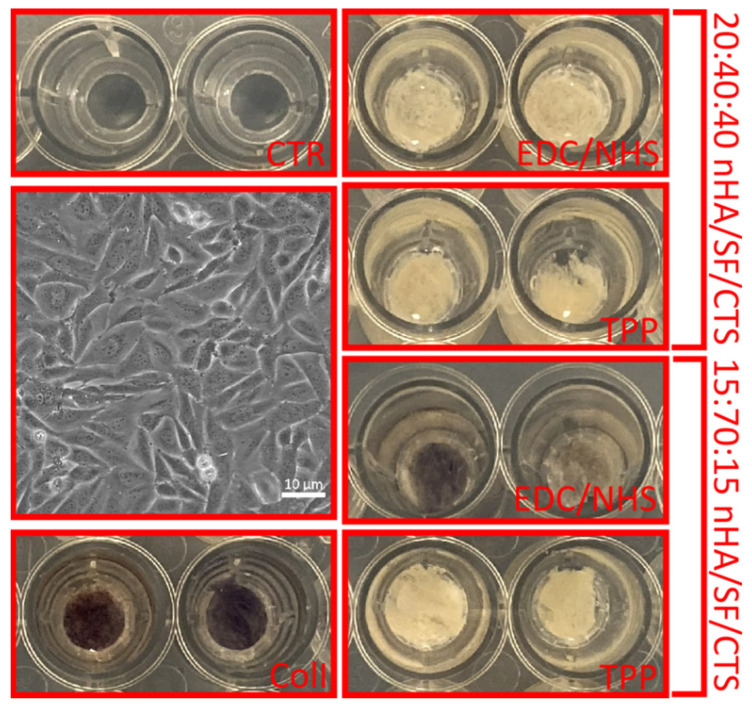
Multi-well culture plate with tested biomaterials after 7 days of inoculation with SaOS-2 cells. Cells from the Control group (CTR, Thermanox™) and experimental groups, including ColI and 20:40:40 or 15:70:15 nHA/SF/CTS scaffolds cross-linked with EDC/NHS or TPP, were incubated with the MTS reagent for 1 h. The formazan staining intensity, clearly visible in the ColI group, reflected the metabolic activity of the cells. The development of the SaOS-2 population in the Control group was monitored by phase-contrast microscopy (scale bar: 10 µm).

**Figure 10:**
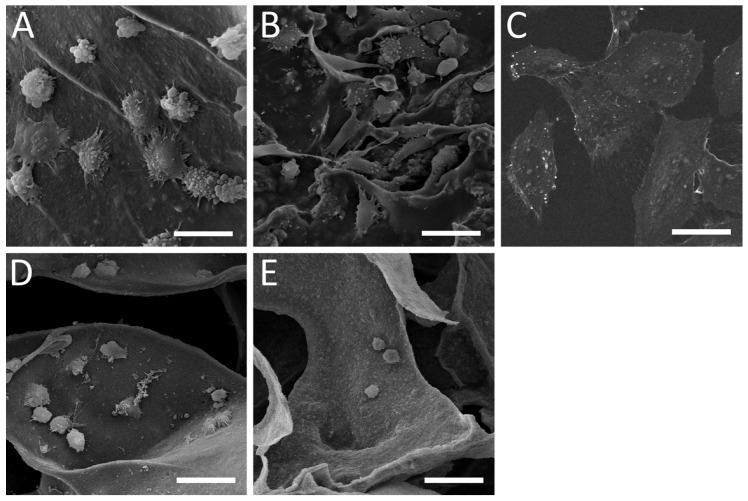
Scanning electron microscopy images of SaOS-2 cells cultured on nHA/SF/CTS scaffolds and the control surface: (**A**) 20:40:40 EDC/NHS, (**B**) 20:40:40 TPP, (**C**) Thermanox™ control, (**D**) 15:70:15 EDC/NHS, and (**E**) 15:70:15 TPP. Scale bars: 25 μm in (**A**,**C**,**E**), and 35 μm in (**B**,**D**). The ColI material is not shown because of its instability and handling difficulties during SEM sample preparation.

**Figure 11:**
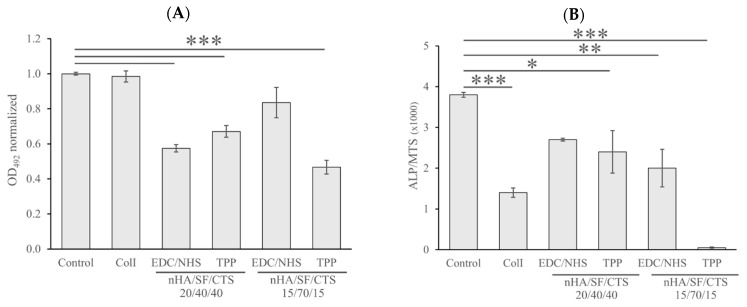
Quantitative evaluation of the biological response of SaOS-2 cells cultured on Control (Thermanox™), ColI, and both nHA/SF/CTS scaffolds—20:40:40 and 15:70:15 cross-linked with EDC/NHS or TPP. (**A**) MTS assay used to assess cell metabolic activity as an indirect indicator of viable cell presence on the tested materials. (**B**) Alkaline phosphatase (ALP) activity used to assess the osteogenic potential of the cells. ALP values were normalized to the corresponding MTS results. Statistical significance was determined by one-way ANOVA followed by Tukey’s HSD post hoc test (* *p* < 0.05, ** *p* < 0.01, *** *p* < 0.001).

To complement the preceding physicochemical characterization with a biologically relevant evaluation, in vitro studies were performed using human osteosarcoma cells (SaOS-2) on both 20:40:40 and 15:70:15 (*w*/*w*) nHA/SF/CTS scaffold compositions. Those compositions were selected for biological assessment because the preceding physicochemical analyses indicated that these materials were suitable for cell-based studies. Thermanox™ (with the compressive modulus (E) value around 2.5 GPa) was used as the reference surface (Control) because it provides a well-established substrate for cell adhesion, growth, and differentiation. Since the ColI and nHA/SF/CTS materials were thick and opaque to visible light, optical microscopy was impractical for these groups, unlike for Thermanox™ (Figure 9). Thus, the samples of the experimental biomaterials were prepared for observation in scanning electron microscopy (SEM) to visualize the morphology of the model cells and their distribution in the scaffold (Figure 10). The MTS assay was used to estimate the metabolic activity of SaOS-2 cells and, indirectly, the extent of scaffold colonization. Representative macroscopic images of the biomaterials in the culture plate after MTS staining are shown in Figure 9. Differences in color intensity suggested differences in the amount of metabolically active cells among the tested materials, which were subsequently evaluated by absorbance measurements. SEM observations (Figure 10) showed that SaOS-2 cells adhered to both EDC/NHS- and TPP-cross-linked nHA/SF/CTS (20:40:40) scaffolds as well as to the Thermanox™ control. However, weaker adhesion was observed on the EDC/NHS-cross-linked nHA/SF/CTS (15:70:15) scaffold. On the TPP-cross-linked 15:70:15 composite, the cells were predominantly rounded and showed the weakest adhesion among the tested scaffolds. Moreover, SEM investigations of the materials revealed that SaOS-2 cells were unevenly distributed within the scaffolds and were mainly localized in biomaterial cavities, while some open surface regions remained devoid of cells. Whereas SaOS-2 cells cultured on Thermanox™ adopted a flattened, well-extended morphology typical of osteoblast-like cells, those observed on the scaffolds were more rounded, with numerous filopodia and other cytoplasmic protrusions (Figure 10). Cell morphology in hydrated three-dimensional scaffold systems reflects the combined influence of adhesion, local surface properties, and scaffold architecture [91]. SaOS-2 cells spread more readily on the flat and homogeneous Thermanox™ surface than on the porous nHA/SF/CTS scaffolds. This difference is consistent with the distinct character of the control surface and the scaffold matrices rather than with stiffness alone. The weakest cell spreading was observed for the TPP-cross-linked 15:70:15 nHA/SF/CTS scaffold, which also showed a less favorable low-strain compressive response after swelling. It is also important to emphasize that the Thermanox™ surface is chemically and physically homogeneous, while the surfaces of the nHA/SF/CTS mixtures must be considered heterogeneous. Therefore, the round shape of the cells with many projections observed in SEM reflects cellular adaptation to local architecture and physicochemical properties of the surface matrix [92]. In the present study, the SEM observations were consistent with the MTS data, indicating that both scaffold types supported viable SaOS-2 populations despite their less uniform colonization relative to the control material (Figure 11A). MTS indicated the highest metabolic activity and extensive colonization of both Thermanox™ and ColI surfaces, whereas viable cell populations were also detected in all scaffold variants, although their number was lower and less uniform throughout the scaffolds. These differences are consistent with the distinct biological roles of the tested materials. ColI provides a favorable substrate for cell attachment because it contains integrin-recognition motifs, including GFOGER, that engage collagen-binding integrins, promote adhesion-related signaling, and support cell spreading and osteogenic differentiation [93,94,95,96]. In contrast, chitosan and silk fibroin do not offer the same repertoire of cell-adhesive motifs, which may contribute to the lower adhesion and less uniform surface colonization observed for the nHA/SF/CTS scaffolds (Figure 11A) [97,98].

To assess the ability of scaffolds to induce osteogenesis, alkaline phosphatase (ALP) activity was measured. The results showed that both 20:40:40 EDC/NHS- and TPP-cross-linked nHA/SF/CTS scaffolds exhibited higher ALP activity than ColI after normalization to the corresponding MTS values (Figure 11B). Similar pro-osteogenic properties were observed for the 15:70:15 EDC/NHS-cross-linked nHA/SF/CTS material, whereas lower ALP activity was recorded for cells cultured on the 15:70:15 nHA/SF/CTS scaffold cross-linked with TPP. In the biologically evaluated scaffolds, silk fibroin and chitosan were present in different proportions, i.e., 20:40:40 and 15:70:15 (*w*/*w*) of nHA/SF/CTS. Previous studies have shown that nHA can promote osteogenesis when incorporated into organic scaffold matrices [99,100,101]. Thus, the higher ALP activity in relation to ColI observed for the nHA-containing scaffolds is consistent with the osteo-supportive contribution of the mineral phase (Figure 11B). Together with the SEM observations, these findings suggest that the less uniform colonization of the 20:40:40 scaffolds, with cells mainly located within pores, did not prevent osteogenic activity. In fully hydrated materials, pores may provide local sites where cells accumulate and interact both with the scaffold and with neighboring cells. Such osteoblastogenesis has been observed in preosteoblasts cultured in the spheroid form [102]. However, cells on the TPP-cross-linked 15:70:15 material showed limited pro-osteogenic activity, consistent with poor SaOS-2 adhesion to this scaffold. Based on studies related to mesenchymal stem cell differentiation into osteoblasts, low material stiffness and limited cell-adherence-promoting surface cues may reduce the activity of Yes-Associated Protein (YAP) and Transcriptional Coactivator with PDZ-binding Motif (TAZ), which are involved in the transduction of mechanical signals to the nucleus and the subsequent reorganization of cellular metabolism toward osteoblastogenesis [103]. Nevertheless, the present results indicate that the 20:40:40 nHA/SF/CTS scaffolds are promising materials for bone-related in vitro applications. Their relatively favorable mechanical profile may also be advantageous for handling and initial structural stability in bone tissue engineering [104]. At the same time, the present results clearly show that these materials do not support cell adhesion and colonization as effectively as the control (Thermanox™) surfaces.

In the present study, SaOS-2 cells were used as an osteoblast-like model to evaluate the initial biological response to the investigated scaffolds [105]. The resulting data provide an initial in vitro assessment based on a single osteoblast-like cell model. SaOS-2 cells exhibit several osteogenic features and are commonly used in biomaterial studies for preliminary evaluation of cell adhesion, metabolic activity, and osteogenic response [106,107]. Under osteogenic culture conditions based on dexamethasone, β-glycerophosphate, and ascorbate-based supplementation, SaOS-2 cells can display osteoblast-like responses, including ALP-associated differentiation and mineralization-related activity, although the outcome depends on the applied supplement combination and the cellular model [108,109]. The response of less differentiated osteo-derived cell lines, such as MG-63 or hFOB, may differ from that observed for SaOS-2 cells because cell adhesion, proliferation, and osteogenic activity are strongly dependent on the cellular model and culture conditions [107,110,111,112]. Primary cells, especially bone marrow-derived stem or progenitor cells, would provide a more physiologically relevant model for verifying the osteogenic response observed for the 20:40:40 nHA/SF/CTS scaffolds [112]. Among the tested materials, the 20:40:40 nHA/SF/CTS scaffold showed increased ALP activity, supporting its further evaluation in more advanced osteogenic models. Related studies on SF/CTS- and SF/CTS/nHA-based systems support this direction of further development. Lai et al. reported that SF and CTS nanofibers supported hMSC proliferation and osteogenic differentiation [113]. Zeng et al. showed that a highly porous SF/CTS mixture (40:60) cross-linked with EDC/NHS was colonized by MG-63 cells and supported adhesion, proliferation, ALP activity, and mineralization [114]. Zhou et al. tested SF/CTS/nHA scaffolds cross-linked with EDC/NHS and reported favorable BMSC adhesion, proliferation, and differentiation when the scaffold was combined with concentrated autologous growth factors. In the same study, implantation in rabbits supported bone-defect repair, as shown by immunohistochemical analysis and microcomputed tomography [22]. Since SaOS-2 adhesion and osteogenic differentiation are sensitive to material-surface properties, further optimization of nHA/SF/CTS scaffold surfaces may improve early cell attachment and scaffold colonization [115]. Further studies using primary osteogenic cells, mesenchymal stem cells, more advanced three-dimensional co-culture systems, bone-on-a-chip platforms, and in vivo models would provide a broader and more translationally relevant assessment of the bone-regenerative potential of these scaffolds [1,106].

## 4. Conclusions

Chemical cross-linking plays a crucial role in improving the structural stability and functional performance of silk fibroin (SF)- and chitosan (CTS)-based materials intended for biomedical applications. The choice of cross-linking agent should therefore be considered not only in terms of scaffold stabilization but also with regard to its effects on material architecture, degradation behavior, mechanical response, and biological performance. Available predicted hazard estimates, considered together with regulatory information from the ECHA database, also indicate TPP as a comparatively low-hazard cross-linking reagent relative to EDC, which is relevant when selecting stabilizing agents for biopolymer-based scaffolds.

In this work, nHA/SF/CTS porous composites with two weight ratios, 20:40:40 and 15:70:15, were successfully fabricated and cross-linked with either EDC/NHS or TPP. The cross-linking route was associated with qualitative differences in scaffold morphology and with differences in swelling capacity, degradation rate, and compressive properties. In general, the 20:40:40 scaffolds showed a more favorable balance of structural stability and mechanical performance than the 15:70:15 variants, although the final effect depended on both scaffold composition and cross-linking chemistry.

Biological studies showed that the 20:40:40 variants obtained using both cross-linking strategies supported SaOS-2 viability and maintained osteogenic activity, whereas the 15:70:15 TPP-cross-linked scaffold showed less favorable biological performance. Although the materials supported cell adhesion and colonization less effectively than ColI or Thermanox™, the observed cell metabolism and ALP activity indicate that selected nHA/SF/CTS scaffolds maintained bone-related cellular activity under the applied in vitro conditions.

The combined results indicate that both EDC/NHS and TPP can be used to prepare nHA/SF/CTS scaffolds, but their suitability for bone-related applications depends strongly on scaffold composition and the target properties. In particular, the 20:40:40 formulation provided the most balanced platform, as both cross-linking routes supported SaOS-2 viability and maintained osteogenic activity, while EDC/NHS provided the highest degradation stability and the most favorable low-strain mechanical response in the 20:40:40 formulation. By contrast, the 15:70:15 formulation showed less favorable performance, particularly after TPP cross-linking, indicating that the fibroin-rich composition was more sensitive to the selected cross-linking route. These findings show that the selection of a cross-linking system should account for both reagent characteristics and their effects on scaffold architecture, swelling, degradation, mechanical behavior, and cell-material interactions. Further optimization of these materials may include tuning their surface characteristics or incorporating additional bioactive cues to promote earlier cell attachment and more homogeneous colonization while preserving the favorable scaffold stability and osteo-supportive behavior observed in the present in vitro model.

## Figures and Tables

**Table 1:** Summary of selected toxicological and hazard-related properties of EDC and TPP. Data were compiled using OECD QSAR Toolbox v.4.5 [47] and publicly available information from the European Chemicals Agency database [48].

Parameter	Compound	
	TPP (CAS: 7758-29-4)	EDC (CAS: 25952-53-8)
Acute toxicity	oral (lack of toxicity)	no data
Eye Irritation	lack of irritation (in vivo, OECD 405)	lack of irritation (in vitro: OECD 437; HET-CAM) GHS criteria not met
Skin Sensitization	OECD 429: negative	OECD 429: positive
Genetic Toxicity	in vitro and in vivo lack of genotoxicity	in vitro: positive (mutagenicity and chromosome aberration study in mammalian cells) in vivo: negative
Repeated dose toxicity	2-year, oral, rat: NOEL = 0.5% in diet lack of CMR effects	reprotoxicity (OECD 422) NOEL = 100 mg/kg/per day

**Table 2:** Summary of low-strain compressive parameters, including compressive modulus (E) and compressive stress at 1% strain (σ_1%_), for dry and PBS-tested nHA/SF/CTS scaffolds.

Scaffold Group	Dry Scaffolds		Scaffolds Tested in PBS (pH 7.4, 37 °C)	
	E [MPa]	σ_1%_ [MPa]	E [kPa]	σ_1%_ [kPa]
20:40:40 EDC/NHS	3.354 ± 0.648	0.0396 ± 0.0081	30.08 ± 2.91	0.961 ± 0.834
20:40:40 TPP	0.787 ± 0.218	0.0208 ± 0.0059	18.54 ± 6.64	0.645 ± 0.231
15:70:15 EDC/NHS	0.730 ± 0.492	0.0221 ± 0.0090	14.18 ± 2.95	0.494 ± 0.077
15:70:15 TPP	0.547 ± 0.079	0.0119 ± 0.0008	9.23 ± 4.15	0.475 ± 0.593

## Data Availability

The original contributions presented in this study are included in the article/Supplementary Materials. Further inquiries can be directed to the corresponding authors.

## Supplementary Materials

The following supporting information can be downloaded at: https://www.mdpi.com/article/10.3390/polym18131610/s1, Figure S1: Compressive stress–strain curves of nHA/SF/CTS 20:40:40 composite scaffolds cross-linked with EDC/NHS tested under dry conditions; Figure S2: Compressive stress–strain curves of nHA/SF/CTS 20:40:40 composite scaffolds cross-linked with TPP tested under dry conditions; Figure S3: Compressive stress–strain curves of nHA/SF/CTS 15:70:15 composite scaffolds cross-linked with EDC/NHS tested under dry conditions; Figure S4: Compressive stress–strain curves of nHA/SF/CTS 15:70:15 composite scaffolds cross-linked with TPP tested under dry conditions; Figure S5: Compressive stress–strain curves of nHA/SF/CTS 20:40:40 composite scaffolds cross-linked with EDC/NHS in a solution of PBS (pH 7.4, 37 °C); Figure S6: Compressive stress–strain curves of nHA/SF/CTS 20:40:40 composite scaffolds cross-linked with TPP in a solution of PBS (pH 7.4, 37 °C); Figure S7: Compressive stress–strain curves of nHA/SF/CTS 15:70:15 composite scaffolds cross-linked with EDC/NHS in a solution of PBS (pH 7.4, 37 °C); Figure S8: Compressive stress–strain curves of nHA/SF/CTS 15:70:15 composite scaffolds cross-linked with TPP in a solution of PBS (pH 7.4, 37 °C).

## References

[1] Nguyen Q.C., Murti B.T., Dong G.C., Huang S.M., Putri A.D., Chen Y.H., Chen C.Y., Yang P.K. (2026). In Vitro Platforms in Bone Tissue Engineering: From Biological Foundations to Advanced Models. ACS Biomater. Sci. Eng..

[2] Grabska-Zielińska S. (2024). Cross-Linking Agents in Three-Component Materials Dedicated to Biomedical Applications: A Review. Polymers.

[3] Krishna V.S., Subashini V., Hariharan A., Chidambaram D., Raaju A., Gopichandran N., Nanthanalaxmi M.P., Lekhavadhani S., Shanmugavadivu A., Selvamurugan N. (2024). Role of crosslinkers in advancing chitosan-based biocomposite scaffolds for bone tissue engineering: A comprehensive review. Int. J. Biol. Macromol..

[4] (2025). Biological Evaluation of Medical Devices—Part 1: Requirements and General Principles for the Evaluation of Biological Safety within a Risk Management Process.

[5] Wang W., Huang W.-C., Zheng J., Xue C., Mao X. (2023). Preparation and comparison of dialdehyde derivatives of polysaccharides as cross-linking agents. Int. J. Biol. Macromol..

[6] Gough J.E., Scotchford C.A., Downes S. (2002). Cytotoxicity of glutaraldehyde crosslinked collagen/poly(vinyl alcohol) films is by the mechanism of apoptosis. J. Biomed. Mater. Res..

[7] Alavarse A.C., Frachini E.C.G., da Silva R.L.C.G., Lima V.H., Shavandi A., Petri D.F.S. (2022). Crosslinkers for polysaccharides and proteins: Synthesis conditions, mechanisms, and crosslinking efficiency, a review. Int. J. Biol. Macromol..

[8] Ahmad Z., Shepherd J.H., Shepherd D.V., Ghose S., Kew S.J., Cameron R.E., Best S.M., Brooks R.A., Wardale J., Rushton N. (2015). Effect of 1-ethyl-3-(3-dimethylaminopropyl) carbodiimide and N-hydroxysuccinimide concentrations on the mechanical and biological characteristics of cross-linked collagen fibres for tendon repair. Regen. Biomater..

[9] Schyra P., Aibibu D., Sundag B., Cherif C. (2025). Chemical Crosslinking of Acid Soluble Collagen Fibres. Biomimetics.

[10] Sionkowska A., Kulka-Kamińska K., Brudzyńska P., Lewandowska K., Piwowarski Ł. (2024). The Influence of Various Crosslinking Conditions of EDC/NHS on the Properties of Fish Collagen Film. Mar. Drugs.

[11] Powell H.M., Boyce S.T. (2006). EDC cross-linking improves skin substitute strength and stability. Biomaterials.

[12] Pańczyszyn E., Jaśko M., Miłek O., Niedziela M., Męcik-Kronenberg T., Hoang-Bujnowicz A., Zięba M., Adamus G., Kowalczuk M., Osyczka A.M. (2021). Gellan gum hydrogels cross-linked with carbodiimide stimulates vacuolation of human tooth-derived stem cells in vitro. Toxicol. Vitr..

[13] Agnes C.J., Karoichan A., Tabrizian M. (2023). The Diamond Concept Enigma: Recent Trends of Its Implementation in Cross-linked Chitosan-Based Scaffolds for Bone Tissue Engineering. ACS Appl. Bio Mater..

[14] Dudeja I., Mankoo R.K., Singh A., Kaur J. (2023). Citric acid: An ecofriendly cross-linker for the production of functional biopolymeric materials. Sustain. Chem. Pharm..

[15] Sapuła P., Bialik-Wąs K., Malarz K. (2023). Are Natural Compounds a Promising Alternative to Synthetic Cross-Linking Agents in the Preparation of Hydrogels?. Pharmaceutics.

[16] Bugnicourt L., Ladavière C. (2016). Interests of chitosan nanoparticles ionically cross-linked with tripolyphosphate for biomedical applications. Prog. Polym. Sci..

[17] Szafrańska J.O., Sołowiej B.G. (2020). Cheese sauces: Characteristics of ingredients, manufacturing methods, microbiological and sensory aspects. J. Food Process Eng..

[18] Vollmer A.H., Kieferle I., Pusl A., Kulozik U. (2021). Effect of pentasodium triphosphate concentration on physicochemical properties, microstructure, and formation of casein fibrils in model processed cheese. J. Dairy Sci..

[19] Gierszewska M., Ostrowska-Czubenko J. (2016). Chitosan-based membranes with different ionic crosslinking density for pharmaceutical and industrial applications. Carbohydr. Polym..

[20] Hidaka M., Kojima M., Sakai S., Delattre C. (2024). Characterization of Chitosan Hydrogels Obtained through Phenol and Tripolyphosphate Anionic Crosslinking. Polymers.

[21] Zeng W., Hui H., Liu Z., Chang Z., Wang M., He B., Hao D. (2021). TPP ionically cross-linked chitosan/PLGA microspheres for the delivery of NGF for peripheral nerve system repair. Carbohydr. Polym..

[22] Zhou Y., Liu X., She H., Wang R., Bai F., Xiang B. (2022). A silk fibroin/chitosan/nanohydroxyapatite biomimetic bone scaffold combined with autologous concentrated growth factor promotes the proliferation and osteogenic differentiation of BMSCs and repair of critical bone defects. Regen. Ther..

[23] Tuwalska A., Sionkowska A., Bryła A., Tylko G., Osyczka A.M., Laus M., Vojtová L. (2022). A Biological Study of Composites Based on the Blends of Nanohydroxyapatite, Silk Fibroin and Chitosan. Materials.

[24] Ye P., Yu B., Deng J., She R.F., Huang W.L. (2017). Application of silk fibroin/chitosan/nano-hydroxyapatite composite scaffold in the repair of rabbit radial bone defect. Exp. Ther. Med..

[25] Wang J., Ma X., Huang P., Zhang X., Zhang Y., Zhao H., Jiao Y., Wang B. (2026). Regulation of bone regeneration by chiral modified hydroxyapatite/chitosan scaffolds. J. Mater. Chem. B.

[26] Czechowska-Biskup R., Jarosińska D., Rokita B., Ulański P., Rosiak J.M. (2012). Determination of degree of deacetylation of chitosan—Comparision of methods. Prog. Chem. Appl. Chitin Its Deriv..

[27] Ostrowska-Czubenko J., Gierszewska-Drużyńska M. (2009). Effect of ionic crosslinking on the water state in hydrogel chitosan membranes. Carbohydr. Polym..

[28] Wu S.-C., Tsou H.-K., Hsu H.-C., Hsu S.-K., Liou S.-P., Ho W.-F. (2013). A hydrothermal synthesis of eggshell and fruit waste extract to produce nanosized hydroxyapatite. Ceram. Int..

[29] Silva C.C., Pinheiro A.G., de Oliveira R.S., Góes J.C., Aranha N., de Oliveira L.R., Sombra A.S.B. (2004). Properties and in vivo investigation of nanocrystalline hydroxyapatite obtained by mechanical alloying. Mater. Sci. Eng. C.

[30] Muralithran G., Ramesh S. (2000). The effects of sintering temperature on the properties of hydroxyapatite. Ceram. Int..

[31] Hossein Fathi M., Mortazavi V., Roohani Esfahani S.I. (2009). Bioactivity Evaluation of Synthetic Nanocrystalline Hydroxyapatite. Dent. Res. J..

[32] Sionkowska A., Tuwalska A. (2020). Preparation and characterization of new materials based on silk fibroin, chitosan and nanohydroxyapatite. Int. J. Polym. Anal. Charact..

[33] Gimenes M.L., Silva V.R., Vieira M.G.A., Silva M.G.C., Scheer A.P. (2014). High Molecular Sericin from Bombyx mori Cocoons: Extraction and Recovering by Ultrafiltration. Int. J. Chem. Eng. Appl..

[34] Park H.J., Lee J.S., Lee O.J., Sheikh F.A., Moon B.M., Ju H.W., Kim J.-H., Kim D.-K., Park C.H. (2014). Fabrication of microporous three-dimensional scaffolds from silk fibroin for tissue engineering. Macromol. Res..

[35] Ajisawa A. (1998). Dissolution of silk fibroin with calcium chloride/ethanol aqueous solution. J. Sericult. Sci. Jpn..

[36] Vojtová L., Pavliňáková V., Muchová J., Kacvinská K., Brtníková J., Knoz M., Lipový B., Faldyna M., Göpfert E., Holoubek J. (2021). Healing and Angiogenic Properties of Collagen/Chitosan Scaffolds Enriched with Hyperstable FGF2-STAB^®^ Protein: In Vitro, Ex Ovo and In Vivo Comprehensive Evaluation. Biomedicines.

[37] Sloviková A., Vojtová L., Jančař J. (2008). Preparation and modification of collagen-based porous scaffold for tissue engineering. Chem. Pap..

[38] Sionkowska A., Płanecka A. (2013). Preparation and characterization of silk fibroin/chitosan composite sponges for tissue engineering. J. Mol. Liq..

[39] Porstmann B., Jung K., Schmechta H., Evers U., Pergande M., Porstmann T., Kramm H.-J., Krause H. (1989). Measurement of lysozyme in human body fluids: Comparison of various enzyme immunoassay techniques and their diagnostic application. Clin. Biochem..

[40] (2002). Plastics—Determination of Compressive Properties.

[41] Osyczka A.M., Nöth U., O’Connor J., Caterson E.J., Yoon K., Danielson K.G., Tuan R.S. (2002). Multilineage Differentiation of Adult Human Bone Marrow Progenitor Cells Transduced with Human Papilloma Virus Type 16 E6/E7 Genes. Calcif. Tissue Int..

[42] Bessey O.A., Lowry O.H., Brock M.J. (1946). A method for the rapid determination of alkaline phosphatase with five cubic millimeters of serum. J. Biol. Chem..

[43] TIBCO Software Inc. (2017). Statistica.

[44] Liu F., Gu Y., Zhao P., Xin H., Gao J., Liu M. (2019). N-hydroxysuccinimide based deep eutectic catalysts as a promising platform for conversion of CO2 into cyclic carbonates at ambient temperature. J. CO2 Util..

[45] Wu S., Wang C., Jin Y., Zhou G., Zhang L., Yu P., Sun L. (2021). Green synthesis of reusable super-paramagnetic diatomite for aqueous nickel (II) removal. J. Colloid Interface Sci..

[46] Li J., Ren N., Qiu J., Jiang H., Zhao H., Wang G., Boughton R.I., Wang Y., Liu H. (2013). Carbodiimide crosslinked collagen from porcine dermal matrix for high-strength tissue engineering scaffold. Int. J. Biol. Macromol..

[47] OECD QSAR Toolbox.

[48] European Chemicals Agency (ECHA) ECHA CHEM, ECHA Chemicals Database. https://chem.echa.europa.eu/.

[49] Zhong J., Zhou X., Ye C., Yu W., Tang Y. (2021). Using FTIR Imaging to Investigate Silk Fibroin-Based Materials. Methods Mol. Biol..

[50] Carissimi G., Baronio C.M., Montalbán M.G., Víllora G., Barth A. (2020). On the Secondary Structure of Silk Fibroin Nanoparticles Obtained Using Ionic Liquids: An Infrared Spectroscopy Study. Polymers.

[51] Zhong J., Liu Y., Ren J., Tang Y., Qi Z., Zhou X., Chen X., Shao Z., Chen M., Kaplan D.L. (2019). Understanding Secondary Structures of Silk Materials via Micro- and Nano-Infrared Spectroscopies. ACS Biomater. Sci. Eng..

[52] Qi Y., Wang H., Wei K., Yang Y., Zheng R.Y., Kim I.S., Zhang K.Q. (2017). A review of structure construction of silk fibroin biomaterials from single structures to multi-level structures. Int. J. Mol. Sci..

[53] Chen X., Shao Z., Marinkovic N.S., Miller L.M., Zhou P., Chance M.R. (2001). Conformation transition kinetics of regenerated Bombyx mori silk fibroin membrane monitored by time-resolved FTIR spectroscopy. Biophys. Chem..

[54] Boulet-Audet M., Vollrath F., Holland C. (2015). Identification and classification of silks using infrared spectroscopy. J. Exp. Biol..

[55] Rinaudo M. (2006). Chitin and chitosan: Properties and applications. Prog. Polym. Sci..

[56] Kumirska J., Czerwicka M., Kaczyński Z., Bychowska A., Brzozowski K., Thöming J., Stepnowski P. (2010). Application of Spectroscopic Methods for Structural Analysis of Chitin and Chitosan. Mar. Drugs.

[57] Atangana E., Chiweshe T.T., Roberts H. (2019). Modification of Novel Chitosan-Starch Cross-Linked Derivatives Polymers: Synthesis and Characterization. J. Polym. Environ..

[58] Sánchez-Machado D.I., López-Cervantes J., Escárcega-Galaz A.A., Campas-Baypoli O.N., Martínez-Ibarra D.M., Rascón-León S. (2024). Measurement of the degree of deacetylation in chitosan films by FTIR, 1H NMR and UV spectrophotometry. MethodsX.

[59] Dimzon I.K.D., Knepper T.P. (2015). Degree of deacetylation of chitosan by infrared spectroscopy and partial least squares. Int. J. Biol. Macromol..

[60] Ren F., Ding Y., Leng Y. (2014). Infrared spectroscopic characterization of carbonated apatite: A combined experimental and computational study. J. Biomed. Mater. Res. Part A.

[61] Fleet M.E. (2009). Infrared spectra of carbonate apatites: ν2-Region bands. Biomaterials.

[62] Silvestro I., Francolini I., Di Lisio V., Martinelli A., Pietrelli L., Scotto d’Abusco A., Scoppio A., Piozzi A. (2020). Preparation and Characterization of TPP-Chitosan Crosslinked Scaffolds for Tissue Engineering. Materials.

[63] Becerra J., Rodriguez M., Leal D., Noris-Suarez K., Gonzalez G. (2022). Chitosan-collagen-hydroxyapatite membranes for tissue engineering. J. Mater. Sci. Mater. Med..

[64] Grabarek Z., Gergely J. (1990). Zero-length crosslinking procedure with the use of active esters. Anal. Biochem..

[65] Rezwan K., Chen Q.Z., Blaker J.J., Boccaccini A.R. (2006). Biodegradable and bioactive porous polymer/inorganic composite scaffolds for bone tissue engineering. Biomaterials.

[66] Collins M.N., Ren G., Young K., Pina S., Reis R.L., Oliveira J.M. (2021). Scaffold Fabrication Technologies and Structure/Function Properties in Bone Tissue Engineering. Adv. Funct. Mater..

[67] Abbasi N., Hamlet S., Love R.M., Nguyen N.-T. (2020). Porous scaffolds for bone regeneration. J. Sci. Adv. Mater. Devices.

[68] Chen X., Li W., Zhong W., Lu Y., Yu T. (1997). pH sensitivity and ion sensitivity of hydrogels based on complex-forming chitosan/silk fibroin interpenetrating polymer network. J. Appl. Polym. Sci..

[69] Xu Z., Tang E., Zhao H. (2019). An Environmentally Sensitive Silk Fibroin/Chitosan Hydrogel and Its Drug Release Behaviors. Polymers.

[70] Aranaz I., Alcántara A.R., Civera M.C., Arias C., Elorza B., Caballero A.H., Acosta N. (2021). Chitosan: An overview of its properties and applications. Polymers.

[71] Gierszewska M., Ostrowska-Czubenko J. (2016). Equilibrium swelling study of crosslinked chitosan membranes in water, buffer and salt solutions. Prog. Chem. Appl. Chitin Its Deriv..

[72] Xiao H., Huang W., Xiong K., Ruan S., Yuan C., Mo G., Tian R., Zhou S., She R., Ye P. (2019). Osteochondral repair using scaffolds with gradient pore sizes constructed with silk fibroin, chitosan, and nano-hydroxyapatite. Int. J. Nanomed..

[73] Degli Esposti M., Changizi M., Salvatori R., Chiarini L., Cannillo V., Morselli D., Fabbri P. (2022). Comparative Study on Bioactive Filler/Biopolymer Scaffolds for Potential Application in Supporting Bone Tissue Regeneration. ACS Appl. Polym. Mater..

[74] Perez R.A., Mestres G. (2016). Role of pore size and morphology in musculo-skeletal tissue regeneration. Mater. Sci. Eng. C.

[75] Jeyachandran D., Cerruti M. (2023). Glass, Ceramic, Polymeric, and Composite Scaffolds with Multiscale Porosity for Bone Tissue Engineering. Adv. Eng. Mater..

[76] Pereira H.F., Cengiz I.F., Silva F.S., Reis R.L., Oliveira J.M. (2020). Scaffolds and coatings for bone regeneration. J. Mater. Sci. Mater. Med..

[77] Ansari M.A.A., Golebiowska A.A., Dash M., Kumar P., Jain P.K., Nukavarapu S.P., Ramakrishna S., Nanda H.S. (2022). Engineering biomaterials to 3D-print scaffolds for bone regeneration: Practical and theoretical consideration. Biomater. Sci..

[78] She Z., Jin C., Huang Z., Zhang B., Feng Q., Xu Y. (2008). Silk fibroin/chitosan scaffold: Preparation, characterization, and culture with HepG2 cell. J. Mater. Sci. Mater. Med..

[79] Zhou T., Wu J., Liu J., Luo Y., Wan Y. (2015). Fabrication and characterization of layered chitosan/silk fibroin/nano-hydroxyapatite scaffolds with designed composition and mechanical properties. Biomed. Mater..

[80] Babu S., Shanmugavadivu A., Selvamurugan N. (2024). Tunable mechanical properties of chitosan-based biocomposite scaffolds for bone tissue engineering applications: A review. Int. J. Biol. Macromol..

[81] Karoyo A.H., Wilson L.D. (2021). A Review on the Design and Hydration Properties of Natural Polymer-Based Hydrogels. Materials.

[82] Cengiz I.F., Pereira H., Espregueira-Mendes J., Kwon I.K., Reis R.L., Oliveira J.M. (2019). Suturable regenerated silk fibroin scaffold reinforced with 3D-printed polycaprolactone mesh: Biomechanical performance and subcutaneous implantation. J. Mater. Sci. Mater. Med..

[83] Yao D., Dong S., Lu Q., Hu X., Kaplan D.L., Zhang B., Zhu H. (2012). Salt-leached silk scaffolds with tunable mechanical properties. Biomacromolecules.

[84] Mandal B.B., Grinberg A., Seok Gil E., Panilaitis B., Kaplan D.L. (2012). High-strength silk protein scaffolds for bone repair. Proc. Natl. Acad. Sci. USA.

[85] Krywka C., Krasnov I., Figuli R., Burghammer M., Müller M. (2014). Determination of silkworm silk fibroin compressibility using high hydrostatic pressure with in situ X-ray microdiffraction. Macromolecules.

[86] Yu Y., Zhang Y., Dai M., Tian Y., Zhang T., Liu Y., Xu J., Wang J. (2026). Regulation of Mineralization and Compressive Properties in Silk Protein Porous Scaffolds to Enhance Osteogenic Differentiation. ACS Biomater. Sci. Eng..

[87] Zhang H., Zhou L., Zhang W. (2014). Control of Scaffold Degradation in Tissue Engineering: A Review. Tissue Eng. Part B Rev..

[88] Echeverria Molina M.I., Malollari K.G., Komvopoulos K. (2021). Design Challenges in Polymeric Scaffolds for Tissue Engineering. Front. Bioeng. Biotechnol..

[89] Luangbudnark W., Viyoch J., Laupattarakasem W., Surakunprapha P., Laupattarakasem P. (2012). Properties and biocompatibility of chitosan and silk fibroin blend films for application in skin tissue engineering. Sci. World J..

[90] Xing X., Han Y., Cheng H. (2023). Biomedical applications of chitosan/silk fibroin composites: A review. Int. J. Biol. Macromol..

[91] Discher D.E., Janmey P., Wang Y.L. (2005). Tissue cells feel and respond to the stiffness of their substrate. Science.

[92] Duarte Campos D.F., Blaeser A., Korsten A., Neuss S., Jäkel J., Vogt M., Fischer H. (2015). The Stiffness and Structure of Three-Dimensional Printed Hydrogels Direct the Differentiation of Mesenchymal Stromal Cells Toward Adipogenic and Osteogenic Lineages. Tissue Eng. Part A.

[93] Sweeney S.M., DiLullo G., Slater S.J., Martinez J., Iozzo R.V., Lauer-Fields J.L., Fields G.B., Antonio J.D.S. (2003). Angiogenesis in Collagen I Requires α2β1 Ligation of a GFP*GER Sequence and Possibly p38 MAPK Activation and Focal Adhesion Disassembly. J. Biol. Chem..

[94] Zheng X., Liu W., Xiang J., Liu P., Ke M., Wang B., Wu R., Lv Y. (2017). Collagen I promotes hepatocellular carcinoma cell proliferation by regulating integrin β1/FAK signaling pathway in nonalcoholic fatty liver. Oncotarget.

[95] Wojtowicz A.M., Shekaran A., Oest M.E., Dupont K.M., Templeman K.L., Hutmacher D.W., Guldberg R.E., García A.J. (2010). Coating of biomaterial scaffolds with the collagen-mimetic peptide GFOGER for bone defect repair. Biomaterials.

[96] Knight C.G., Morton L.F., Peachey A.R., Tuckwell D.S., Farndale R.W., Barnes M.J. (2000). The collagen-binding A-domains of integrins α1β1 and α2β1 recognize the same specific amino acid sequence, GFOGER, in native (triple-helical) collagens. J. Biol. Chem..

[97] Wang L., Stegemann J.P. (2010). Thermogelling chitosan and collagen composite hydrogels initiated with β-glycerophosphate for bone tissue engineering. Biomaterials.

[98] Wang J., Yang Q., Mao C., Zhang S. (2012). Osteogenic differentiation of bone marrow mesenchymal stem cells on the collagen/silk fibroin bi-template-induced biomimetic bone substitutes. J. Biomed. Mater. Res. Part A.

[99] Ma R., Tang S., Tan H., Lin W., Wang Y., Wei J., Zhao L., Tang T. (2014). Preparation, characterization, and in vitro osteoblast functions of a nano-hydroxyapatite/polyetheretherketone biocomposite as orthopedic implant material. Int. J. Nanomed..

[100] Pilloni A., Pompa G., Saccucci M., Di Carlo G., Rimondini L., Brama M., Zeza B., Wannenes F., Migliaccio S. (2014). Analysis of human alveolar osteoblast behavior on a nano-hydroxyapatite substrate: An in vitro study. BMC Oral Health.

[101] Ha S.W., Jang H.L., Nam K.T., Beck G.R. (2015). Nano-hydroxyapatite modulates osteoblast lineage commitment by stimulation of DNA methylation and regulation of gene expression. Biomaterials.

[102] Koblenzer M., Weiler M., Fragoulis A., Rütten S., Pufe T., Jahr H. (2022). Physiological Mineralization during In Vitro Osteogenesis in a Biomimetic Spheroid Culture Model. Cells.

[103] Virdi J.K., Pethe P. (2021). Biomaterials Regulate Mechanosensors YAP/TAZ in Stem Cell Growth and Differentiation. Tissue Eng. Regen. Med..

[104] O’Brien F.J. (2011). Biomaterials & scaffolds for tissue engineering. Mater. Today.

[105] Rodan S.B., Imai Y., Thiede M.A., Wesolowski G., Thompson D., Bar-Shavit Z., Shull S., Mann K., Rodan G.A. (1987). Characterization of a Human Osteosarcoma Cell Line (Saos-2) with Osteoblastic Properties. Cancer Res..

[106] Dvorakova J., Wiesnerova L., Chocholata P., Kulda V., Landsmann L., Cedikova M., Kripnerova M., Eberlova L., Babuska V. (2023). Human cells with osteogenic potential in bone tissue research. Biomed. Eng. Online.

[107] Czekanska E.M., Stoddart M.J., Ralphs J.R., Richards R.G., Hayes J.S. (2014). A phenotypic comparison of osteoblast cell lines versus human primary osteoblasts for biomaterials testing. J. Biomed. Mater. Res. Part A.

[108] Yin X., Chen Z., Liu Z., Song C. (2012). Tissue transglutaminase (TG2) activity regulates osteoblast differentiation and mineralization in the SAOS-2 cell line. Braz. J. Med. Biol. Res..

[109] Yevlashevskaya O.S., Scheven B.A., Walmsley A.D., Shelton R.M. (2023). Differing responses of osteogenic cell lines to β-glycerophosphate. Sci. Rep..

[110] Dedhar S., Mitchell M.D., Pierschbacher M.D. (1989). The osteoblast-like differentiated phenotype of a variant of mg-63 osteosarcoma cell line correlated with altered adhesive properties. Connect. Tissue Res..

[111] Harris S.A., Enger R.J., Riggs B.L., Spelsberg T.C. (1995). Development and characterization of a conditionally immortalized human fetal osteoblastic cell line. J. Bone Miner. Res..

[112] Yuste I., Luciano F.C., González-Burgos E., Lalatsa A., Serrano D.R. (2021). Mimicking bone microenvironment: 2D and 3D in vitro models of human osteoblasts. Pharmacol. Res..

[113] Lai G.-J., Shalumon K.T., Chen S.-H., Chen J.-P. (2014). Composite chitosan/silk fibroin nanofibers for modulation of osteogenic differentiation and proliferation of human mesenchymal stem cells. Carbohydr. Polym..

[114] Zeng S., Liu L., Shi Y., Qiu J., Fang W., Rong M., Guo Z., Gao W. (2015). Characterization of silk fibroin/chitosan 3D porous scaffold and in vitro cytology. PLoS ONE.

[115] Voltrova B., Hybasek V., Blahnova V., Sepitka J., Lukasova V., Vocetkova K., Sovkova V., Matejka R., Fojt J., Joska L. (2019). Different diameters of titanium dioxide nanotubes modulate Saos-2 osteoblast-like cell adhesion and osteogenic differentiation and nanomechanical properties of the surface. RSC Adv..

