# Supplementary material for: Comparison of EDC/NHS and Pentasodium Triphosphate (TPP) as Cross-Linking Agents for Nanohydroxyapatite, Silk Fibroin, and Chitosan 3D Scaffolds
Source: Polymers (Basel). 2026 Jun 28;18(13):1610. doi: 10.3390/polym18131610 (PMC13364012; doi:10.3390/polym18131610)
Supplement: Supplementary file 1 [file polymers-18-01610-s001.zip › polymers-4354786-supplementary.pdf]

Comparison of EDC/NHS and pentasodium triphosphate (TPP) as cross-linking agents for nanohydroxyapatite, silk fibroin, and chitosan 3D scaffolds

Anna Tuwalska [1,*], Alina Sionkowska [1,2,*], Grzegorz Tylko [3], Maciej Przybyłek [2,4], Anna Maria Osyczka [3], Iwona Białas [5] and Michele Laus [6]

[1] Department of Biomaterials and Cosmetics Chemistry, Faculty of Chemistry, Nicolaus Copernicus University in Toruń, 87-100 Toruń, Poland
[2] Institute of Advanced Studies, Nicolaus Copernicus University in Toruń, Wileńska 4, 87-100 Toruń, Poland; m.przybylek@cm.umk.pl
[3] Department of Biology and Cell Imaging, Institute of Zoology and Biomedical Research, Faculty of Biology, Jagiellonian University, 30-387 Kraków, Poland; grzegorz.tylko@uj.edu.pl (G.T.); anna.osyczka@uj.edu.pl (A.M.O.)
[4] Department of Physical Chemistry, Pharmacy Faculty, Collegium Medicum in Bydgoszcz, Nicolaus Copernicus University in Toruń, Kurpińskiego 5, 85-096 Bydgoszcz, Poland
[5] CosmetoSAFE Consulting LLC, 05-500 Piaseczno, Poland; i.bialas@cosmetosafe.pl
[6] Department of Science and Technological Innovation, University of Eastern Piedmont "A. Avogadro", 15121 Alessandria, Italy; michele.laus@uniupo.it
* Correspondence: planecka@doktorant.umk.pl (A.T.); as@chem.umk.pl (A.S.); Tel.: +48-56-611-4547 (A.S.)

Table of Content

Compressive stress-strain curves 2

Figure S1. Compressive stress-strain curves of nHA/SF/CTS 20:40:40 composite scaffolds cross-linked with EDC/NHS tested under dry conditions. 2

Figure S2. Compressive stress-strain curves of nHA/SF/CTS 20:40:40 composite scaffolds cross-linked with TPP tested under dry conditions. 3

Figure S3. Compressive stress-strain curves of nHA/SF/CTS 15:70:15 composite scaffolds cross-linked with EDC/NHS tested under dry conditions. 4

Figure S4. Compressive stress-strain curves of nHA/SF/CTS 15:70:15 composite scaffolds cross-linked with TPP tested under dry conditions. 5

Figure S5. Compressive stress-strain curves of nHA/SF/CTS 20:40:40 composite scaffolds cross-linked with EDC/NHS in a solution of PBS (pH 7.4, 37 °C). .6

Figure S6. Compressive stress-strain curves of nHA/SF/CTS 20:40:40 composite scaffolds cross-linked with TPP in a solution of PBS (pH 7.4, 37 °C). 7

Figure S7. Compressive stress-strain curves of nHA/SF/CTS 15:70:15 composite scaffolds cross-linked with EDC/NHS in a solution of PBS (pH 7.4, 37 °C)..8

Figure S8. Compressive stress-strain curves of nHA/SF/CTS 15:70:15 composite scaffolds cross-linked with TPP in a solution of PBS (pH 7.4, 37 °C). 9

Compressive stress-strain curves

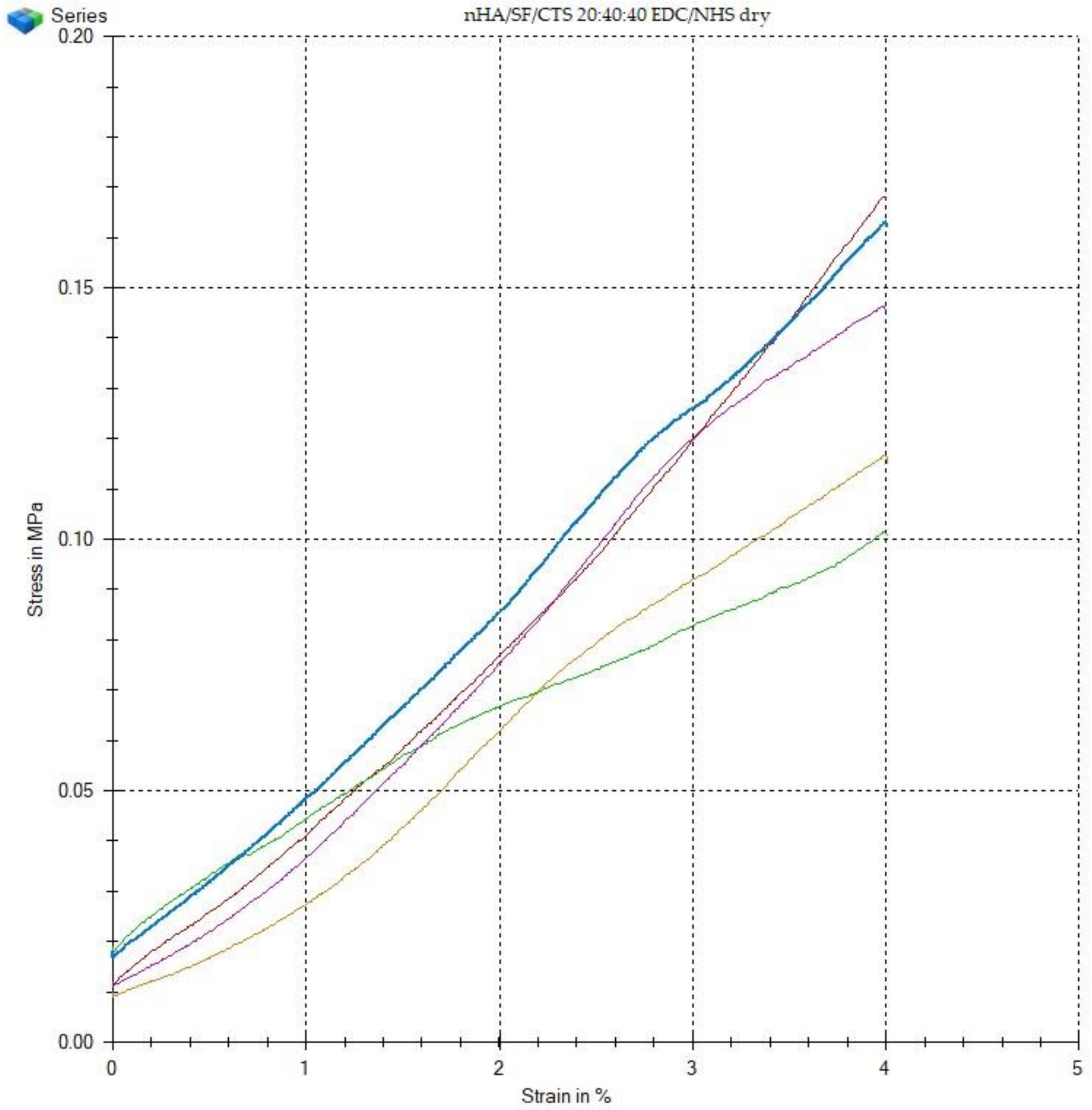

Figure S1. Compressive stress-strain curves of nHA/SF/CTS 20:40:40 composite scaffolds cross-linked with EDC/NHS tested under dry conditions.

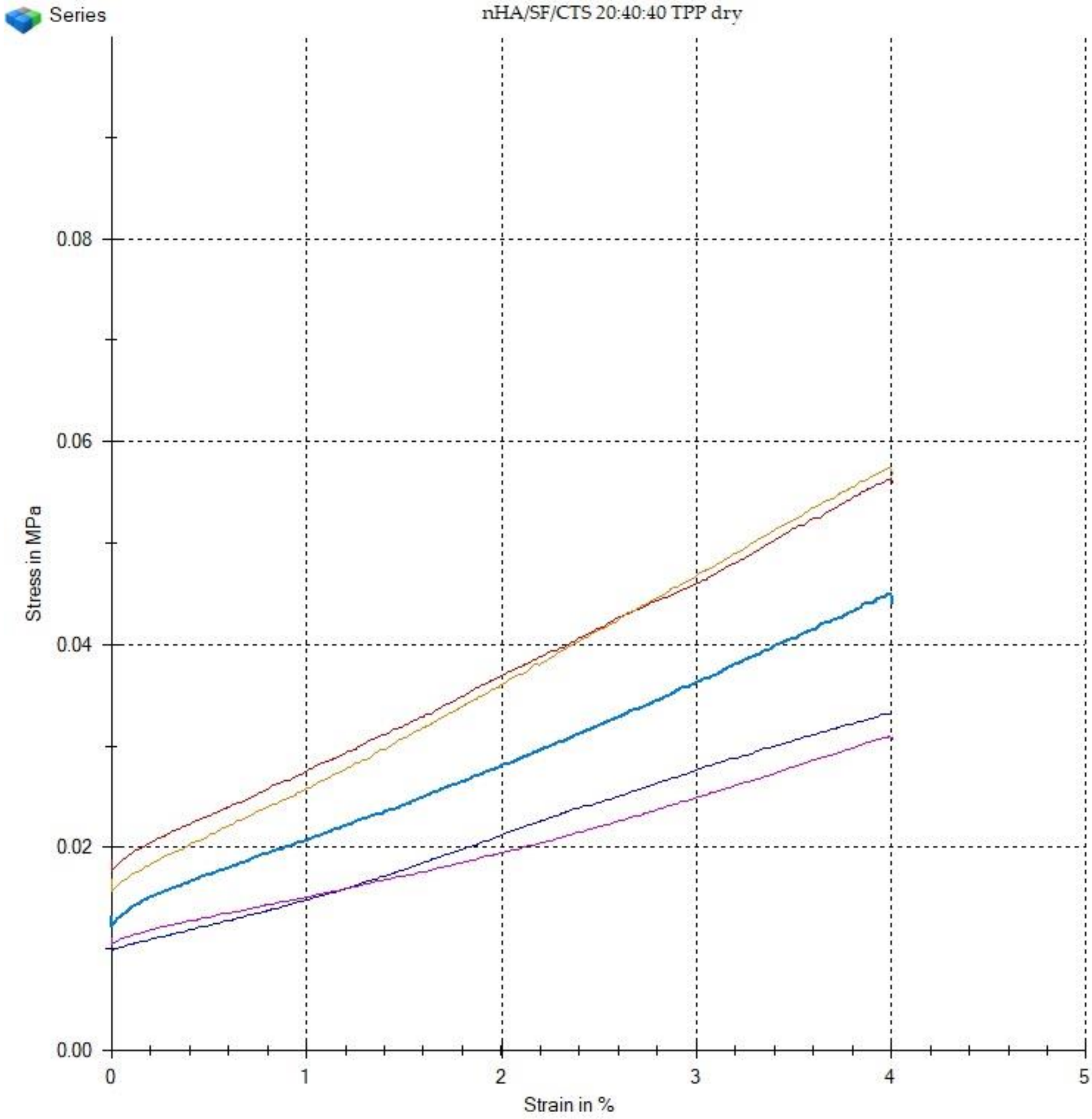

Figure S2. Compressive stress-strain curves of nHA/SF/CTS 20:40:40 composite scaffolds cross-linked with TPP tested under dry conditions.

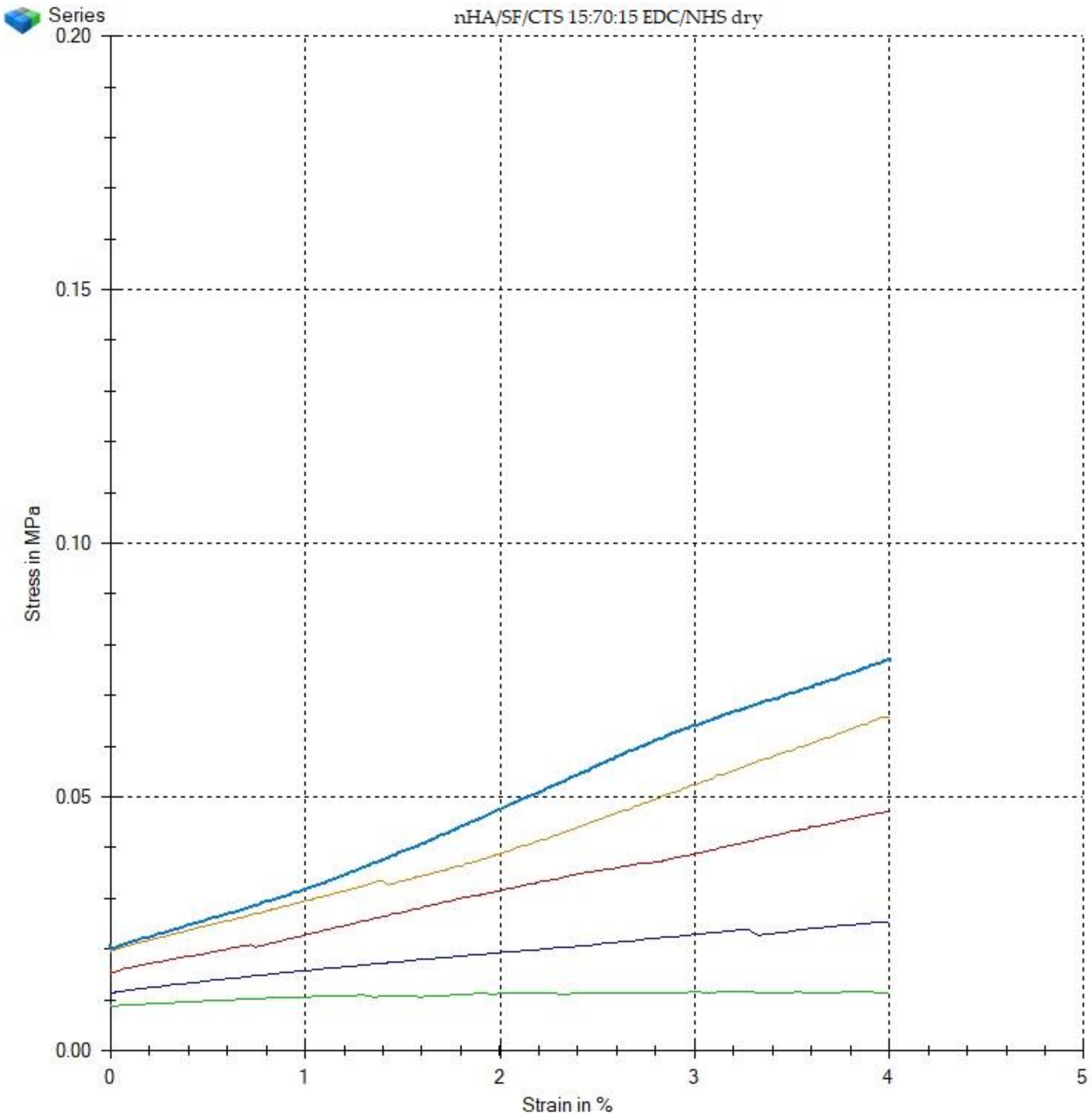

Figure S3. Compressive stress-strain curves of nHA/SF/CTS 15:70:15 composite scaffolds cross-linked with EDC/NHS tested under dry conditions.

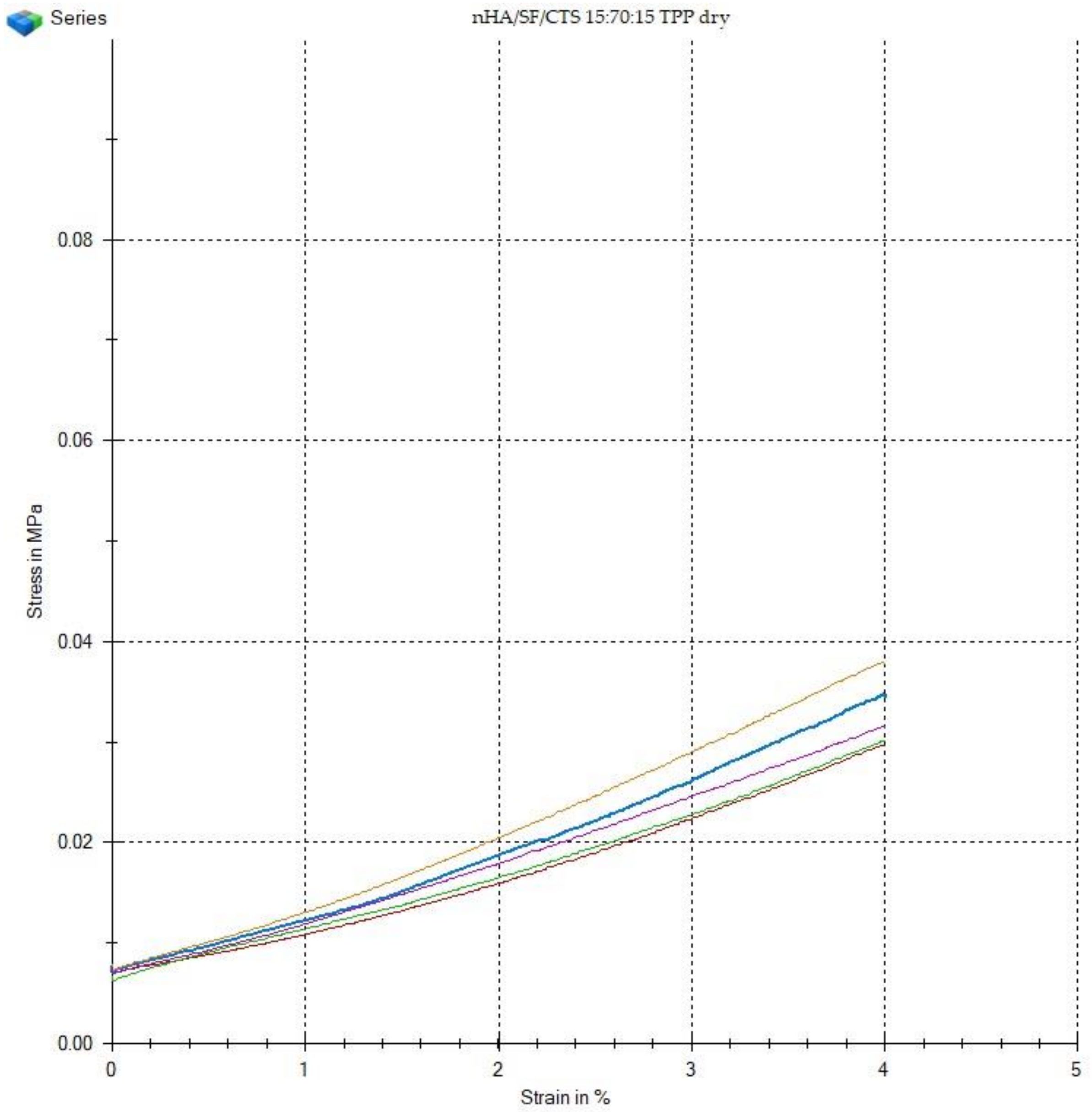

Figure S4. Compressive stress-strain curves of nHA/SF/CTS 15:70:15 composite scaffolds cross-linked with TPP tested under dry conditions.

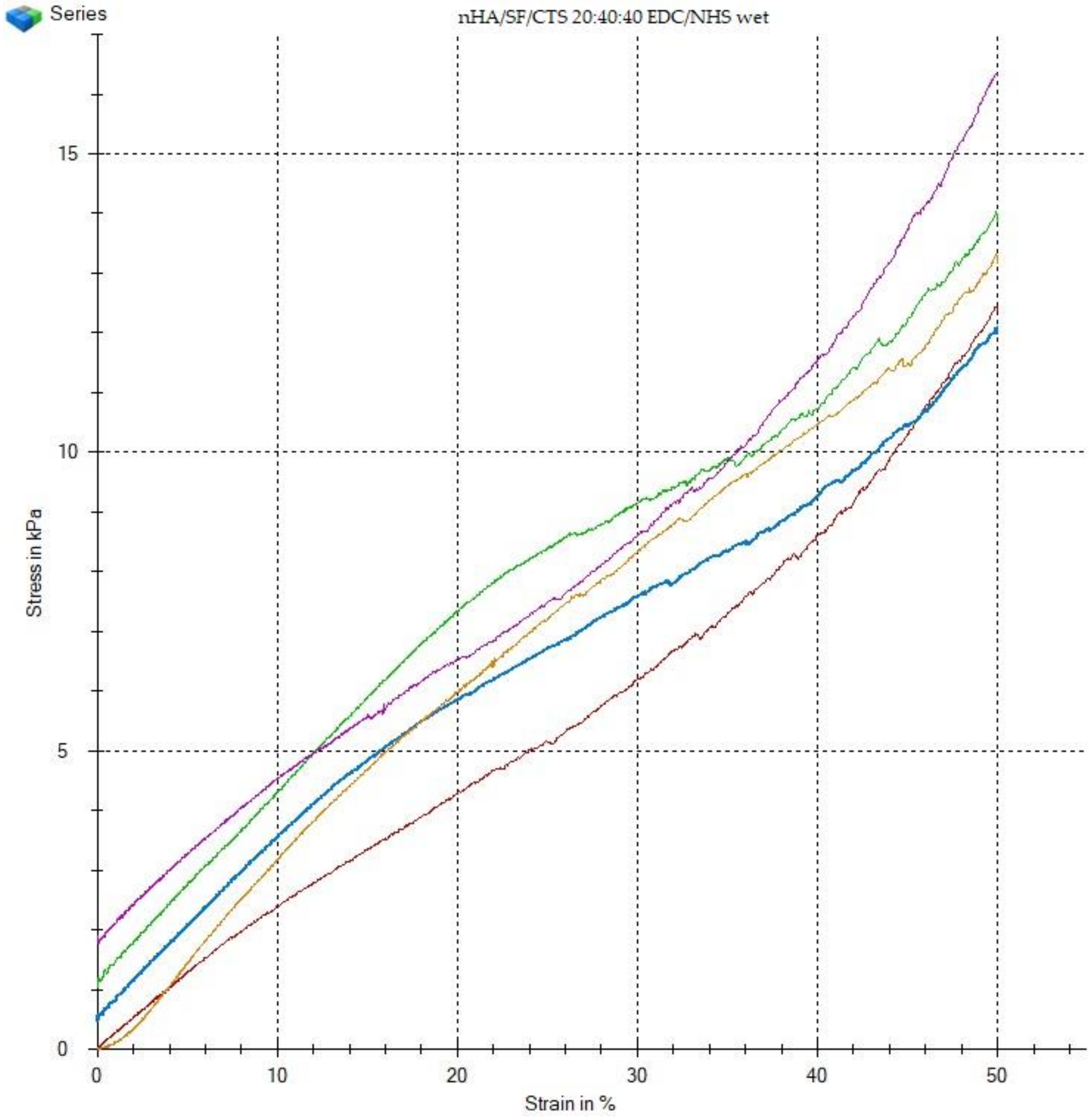

Figure S5. Compressive stress-strain curves of nHA/SF/CTS 20:40:40 composite scaffolds cross-linked with EDC/NHS in a solution of PBS (pH 7.4, 37 °C).

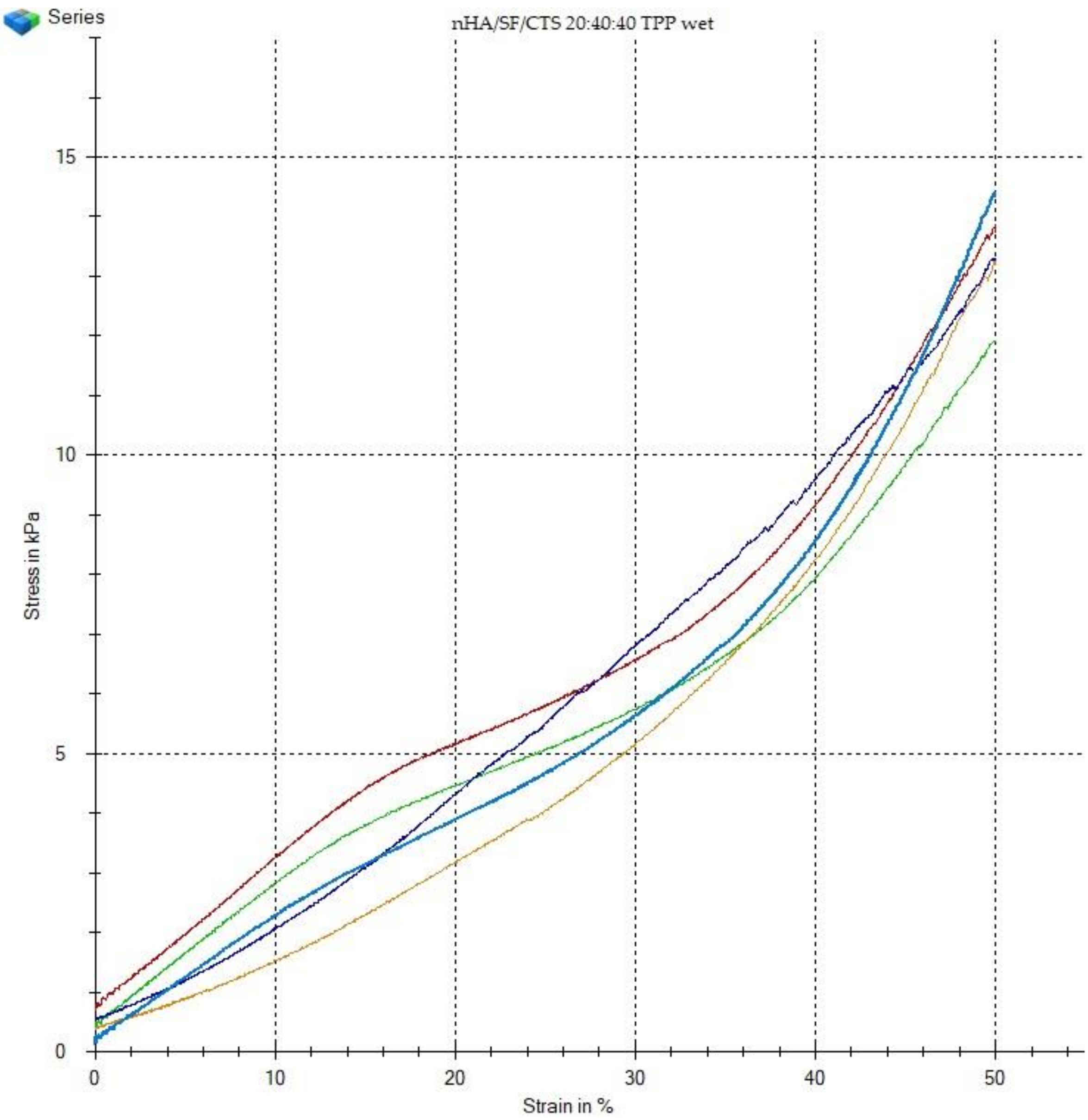

Figure S6. Compressive stress-strain curves of nHA/SF/CTS 20:40:40 composite scaffolds cross-linked with TPP in a solution of PBS (pH 7.4, 37 °C).

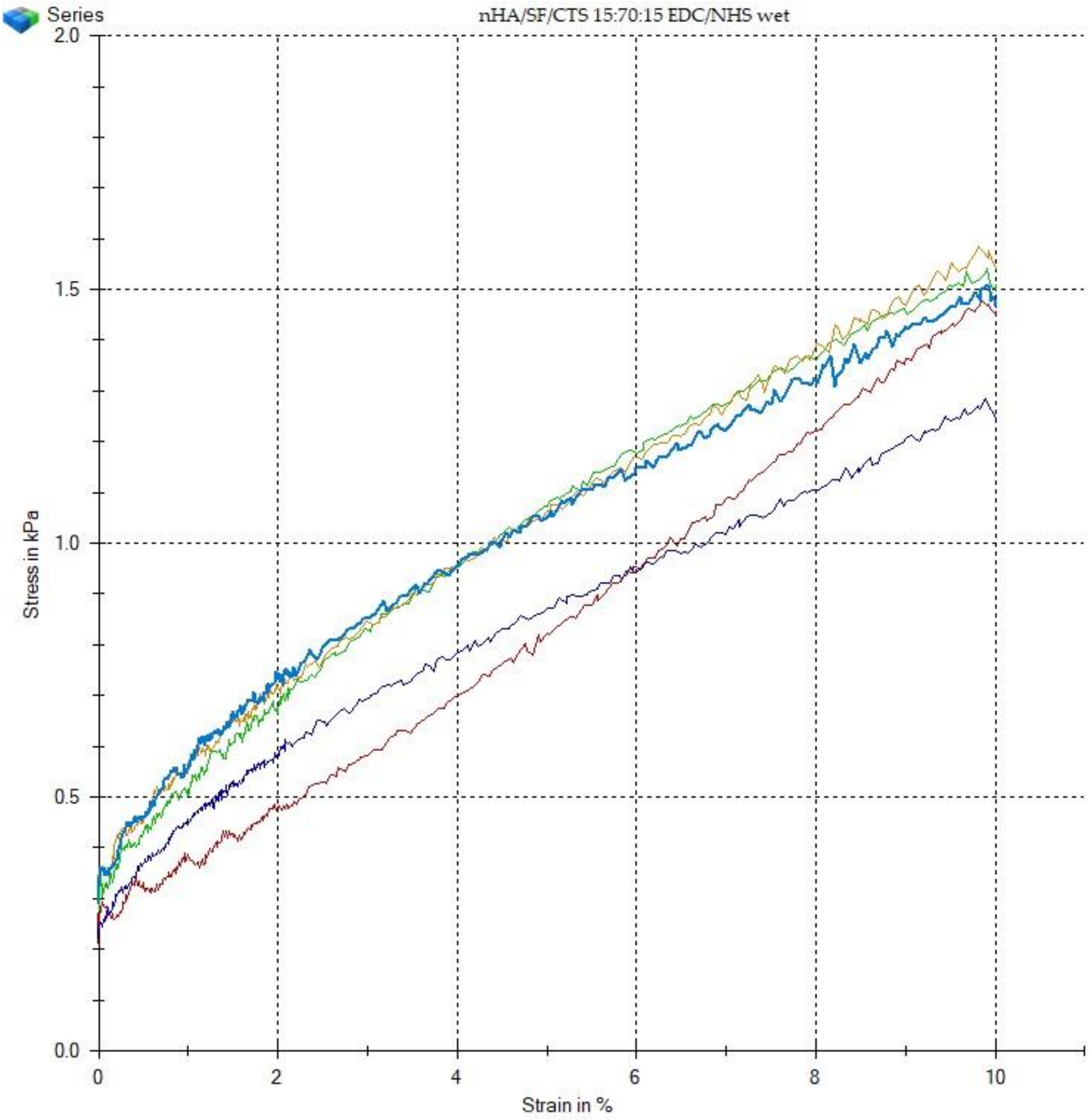

Figure S7. Compressive stress-strain curves of nHA/SF/CTS 15:70:15 composite scaffolds cross-linked with EDC/NHS in a solution of PBS (pH 7.4, 37 °C).

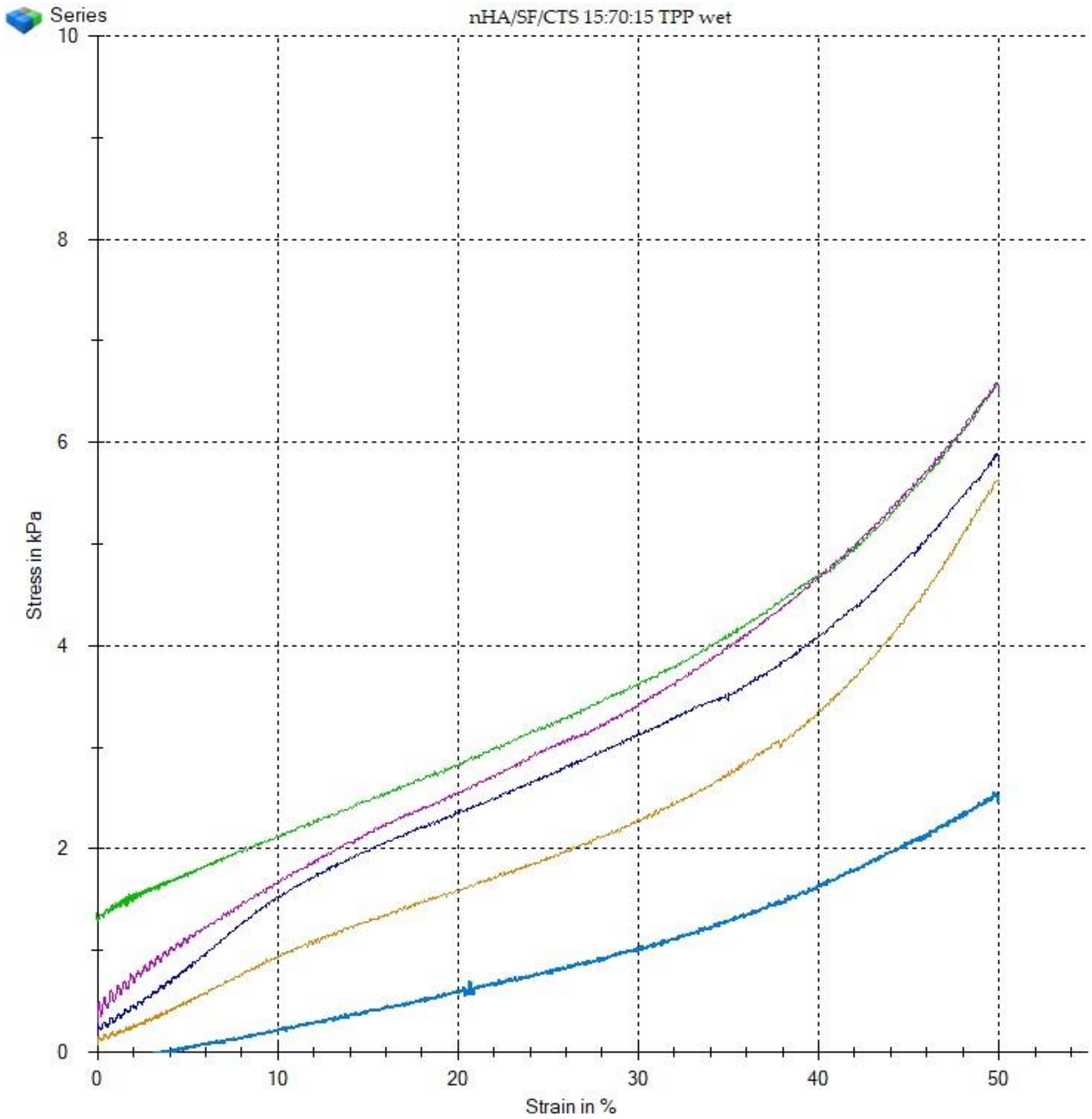

Figure S8. Compressive stress-strain curves of nHA/SF/CTS 15:70:15 composite scaffolds cross-linked with TPP in a solution of PBS (pH 7.4, 37 °C).
